# Gene set enrichment analysis to create polygenic scores: a developmental examination of aggression

**DOI:** 10.1038/s41398-019-0513-7

**Published:** 2019-09-02

**Authors:** Kit K. Elam, Sierra Clifford, Daniel S. Shaw, Melvin N. Wilson, Kathryn Lemery-Chalfant

**Affiliations:** 1 Department of Applied Health Science, Indiana University - Bloomington, Bloomington, IN USA; 2 Department of Psychology, Arizona State University, Tempe, AZ USA; 3 Department of Psychology, University of Pittsburgh, Pittsburgh, PA USA; 4 Department of Psychology, University of Virginia, Charlottesville, VA USA

**Keywords:** Genomics, Human behaviour

## Abstract

Previous approaches for creating polygenic risk scores (PRSs) do not explicitly consider the biological or developmental relevance of the genetic variants selected for inclusion. We applied gene set enrichment analysis to meta-GWAS data to create developmentally targeted, functionally informed PRSs. Using two developmentally matched meta-GWAS discovery samples, separate PRSs were formed, then examined in time-varying effect models of aggression in a second, longitudinal sample of children (*n* *=* 515, 49% female) in early childhood (2–5 years old), and middle childhood (7.5–10.5 years old). Functional PRSs were associated with aggression in both the early and middle childhood models.

## Introduction

A growing literature examines polygenic risk scores (PRSs) formed from multiple single nucleotide polymorphisms (SNPs) as indices of genetic risk^1–8^. To date, two approaches are most commonly used in forming these scores. One approach is theoretically driven, in which a PRS is formed from a small number of candidate SNPs selected from genes proposed to be relevant to a trait or behavior^9^. Another common approach is data-driven, and involves forming a score which typically includes a large number of SNPs drawn from a discovery genome wide association study (GWAS), with all SNPs below a certain significance threshold selected for inclusion^6^. Both approaches have strengths and limitations. The first approach attempts to elucidate biological pathways, but often relies heavily on a few SNPs and candidate genes selected for proposed functional relevance. The second approach leverages the power of GWAS to detect small effects across the genome^10^, including the effects of SNPs in pathways overlooked by candidate gene research. The GWAS approach is blind to theory and it is possible to include large numbers of SNPs in which some associations are driven purely by chance. The strongest approach would therefore integrate theoretical or actual knowledge of biological processes determined from biological databases with statistically selected SNPs^11–13^. Such a biologically informed approach could improve the predictive accuracy of PRSs and explain biological pathways underlying genetic influences on behavior. An additional limitation is that research using both approaches often derives PRSs from findings in an initial discovery sample that is developmentally divergent from the sample currently being tested (e.g., SNPs drawn from a discovery GWAS of adults are used to test a PRS in a childhood sample). This is problematic because genetic influences can be dynamic across the lifespan and vary by physical and socio-cultural environmental conditions^14–17^.

We examined a new approach for creating biologically informed PRSs for aggression across childhood. We first used a traditional data-driven approach to form two PRSs for aggression from separate discovery meta-GWASs in (a) early childhood and (b) middle-to-late childhood^18^. Next, we used a bioinformatics tool, gene set enrichment analysis (GSEA; 11–13), to inform selection of SNPs from the same meta-GWASs of childhood aggression in early and middle childhood, respectively. Using GSEA we created two biologically refined PRSs for each developmental period. These PRSs included: (a) those SNPs that significantly mapped to gene sets, and (b) those SNPs that significantly mapped to gene sets and were functional. Thus, we test the predictive utility of three PRSs for aggression in each developmental period (one traditional PRS and two GSEA-informed PRSs) with aggressive behavior across early childhood (2–5 years of age), and across middle childhood (7.5–10.5 years of age) using separate time-varying effect models.

## Genetic effects on childhood aggression

Genetic associations are typically viewed as static and are tested in samples with wide age ranges. However, seminal genetic and developmental theories propose that genetic effects are developmental in nature and vary over time^14–17^. Hence, there is increasing evidence of developmental genetic effects on aggressive behavior from adoption^19^, twin^20^, and molecular genetic studies^6,21^. Developmental studies illustrate that both the strength of genetic effects, and the relevant genetic variants may vary over the life course based on differences in environmental context and genetic processes. Because genetic factors can account for both stability and change in aggression across childhood, developmentally specific indices of genetic risk for aggression likely capture less variants accounting for stability and would not be expected to replicate across developmental periods^22–24^. However, this developmental specificity has been less examined relative to PRSs.

Although a growing number of studies examine PRSs in adulthood in relation to health outcomes^1^, substance use^2^, and psychiatric outcomes^8^, relatively few studies have examined polygenic risk in childhood. Even fewer have considered aggression or other indices of externalizing behavior during this period, so associated phenotypes are also reviewed. In particular, polygenic risk for ADHD was associated with comorbid ADHD and conduct problems in 6–17 year olds^3^. Another study found that polygenic risk for behavioral disinhibition was associated with behaviorally disinhibited behavior and nicotine, alcohol, and drug use in adolescence^4^. Some studies have found developmental specificity in polygenic scores. Researchers created a PRS for externalizing behavior based on an adult GWAS of externalizing disorder, and found that it was associated more strongly with externalizing behavior in adolescence compared with young adulthood^6^. Other researchers formed a PRS for behavioral disinhibition based on SNPs identified through a review of the literature, including samples spanning from childhood to adulthood^7^. This PRS was associated with impulsivity in middle childhood but not late childhood. Finally, a PRS based on a predominately child-based discovery sample predicted both ADHD symptoms but also general psychopathology^25^. Thus, evidence indicates that PRSs are associated with indices of externalizing behavior and psychopathology. However, these results are primarily in adolescent samples with few studies in childhood and no studies in early childhood. Early studies on polygenic effects on aggression and related indices are informative but are limited in the developmental conclusions that can be drawn.

More broadly, whereas there has been some preliminary examination of developmental specificity of polygenic effects in which SNPs were drawn from adult samples, no study has tested the developmental specificity of PRSs based on GWASs that are developmentally matched to the age in which they were originally tested. In addition to these developmental limitations, the data-driven approach and the theoretical approach to forming PRSs each have their own conceptual and methodological challenges.

## Creation of polygenic risk scores

Two complementary concepts underlie the use of PRSs: the idea that complex traits and behaviors are polygenic or affected by a number of genes, and the idea that these outcomes are influenced in part by pleiotropy, in which each gene affects a number of behaviors^8,10^. This framework and converging evidence from GWASs illustrate that most complex behaviors have an underlying polygenic architecture^10^, which has prompted a recent rise in examination of PRSs in relation to psychopathological outcomes. One method for forming PRSs has been to use a theoretically or hypothesis-driven approach in selecting SNPs from the literature based on known or assumed associations with the trait or behavior of interest; the theoretical derived approach is similar to the candidate gene approach^5^. For example, SNPs have been chosen based on the knowledge that they reside in genes that are broadly related to a certain biological function and the related behavior^26^. For some well-validated genetic effects this is plausible and links to biological processes have been established. However, in other cases the theoretical approach is often predicated on theorized or ambiguous biological relationships between genes and biological processes, with no evidence that SNPs within the proposed genes have any biological function. The theoretical approach also suffers from the limitations associated with candidate gene research, including multiple testing issues, small effects, and a high likelihood of missing meaningful associations^27,28^.

A second common method for generating PRSs uses a data-driven, hypothesis-free approach to selecting SNPs. Specifically, SNPs found to be associated with the outcome of interest in a previous GWAS are combined into a PRS, often composed of hundreds or thousands of SNPs^5^. Commonly, multiple PRSs are formed using different significance thresholds for data-driven SNP selection (e.g., *p* < 0.01, *p* < 0.05, *p* < 0.10…*p* *<* 0.70), and in some cases all SNPs from a GWAS are included in a score, weighted by effect size. Genetic associations between each PRS and a phenotype are then tested, often in one or more separate replication samples, and the best score is considered the one that explains the most variance in the outcome. However, this approach raises concerns about multiple testing and Type I error. Further, forming scores that include data-driven SNPs that are not even nominally associated in the original GWAS (i.e., *p* *>* 0.30 or greater), or including all SNPs from a GWAS likely introduces spurious variance. Such a GWAS-based approach may be of little theoretical value and result in diluting a true signal. Finally, scores formed using this GWAS-based method have no biological relevance. By including SNPs associated with a phenotype based on a broad statistical threshold, it is difficult to make connections to a single, or even multiple biological systems or processes. Indeed, SNPs included in such scores may fall within regions of genes and have no functional relevance at all.

Bioinformatics tools offer the possibility of applying biological relevance to GWAS data in secondary analyses. Many methods for utilizing these tools exist, but one increasingly frequent approach being applied to GWAS data is Gene Set Enrichment Analysis (GSEA; 11–13). A *gene set* is a collection of genes that can represent a biological process (e.g., molecular, cellular, disease), but may also represent gene networks and ontologies. Known gene sets are available from numerous public databases, which specify the genes in each set and the process they represent^13,29,30^. GSEA is a statistical procedures that provides information about which gene sets a given gene, or multiple genes, belong to and what biological processes they represent, based on information accessible from these public databases. For a detailed walkthrough and recommendations for running GSEA, see Mooney and Wilmot^12^.

An innovative variation of GSEA is to statistically test whether SNPs within genes are significantly associated with a gene set. The broad steps involved in this type of GSEA vary based on the software used; the following description is based on iGSEA4GWASv2^31,32^. First, the user typically specifies which gene sets to load from publicly available databases; either all gene sets or only those related to certain databases or processes (e.g., gene ontology). Next, the user provides a list of SNPs and their respective *p*-values from association tests with a given phenotype, such as those from a relevant discovery GWAS. These SNPs are then mapped to genes based on SNP and gene annotations from an online database (e.g., Ensembl Biomart) within the user-specified range upstream and downstream^33^. Each gene is ranked based on the number of SNPs in each gene and their respective *p*-values. These genes (and their ranks) are then compared with the available gene sets to calculate each set’s enrichment score; that is, the proportion of the association between the gene(s) and target gene sets compared with the association between gene(s) outside gene sets^12^. Finally, permutation tests apply a false-discovery rate (FDR) to correct for multiple testing, gene set size, and overlap in gene sets^11,12^. These procedures result in a list of SNPs and their respective gene and the gene set to which each gene was mapped. Thus, using GSEA can be applied to a large group of SNPs from a GWAS to filter and derive a smaller group of SNPs, which map to a gene set. These SNPs can then be formed into a biologically informed PRS.

A recent option in some GSEA software further refines the list of SNPs that were successfully mapped to genes and gene sets to those SNPs (or a SNP in LD proxy) that are functional^32^. Functionality can be conferred by annotation (e.g., a SNP resides in a coding region associated with a protein or RNA product), regulatory regions (e.g., a SNP resides in a region that controls the expression of other coding regions), or eQTL (e.g., a SNP is associated with variation in expression of mRNA or protein). Collectively, GSEA with functional SNP identification can be used to classify SNPs at two levels^1^: those SNPs significantly mapped to a gene set, or^2^ those SNPs both significantly mapped to a gene set and noted as functional.

To date, GSEA is being used with GWAS data to identify specific biological processes involved in disease outcomes in the medical literature (e.g., lung function^34^), with some emerging research on psychopathology outcomes such as ADHD^35^. However, these emerging studies typically examine single SNPs resulting from GSEA or the effect of single pathways. To our knowledge, no study has created PRSs composed of functional SNPs resulting from GSEA. Thus, the current study is the first to create biologically informed PRSs for psychopathological outcomes, by first filtering meta-GWAS data for child aggression through GSEA, then forming PRSs from SNPs that significantly mapped to gene sets and for the subset of SNPs with a known biological function.

An additional strength of the present study is that the discovery meta-GWASs examined associations with child aggression separately in early and middle childhood^18^, allowing us to create separate PRSs targeted to each of the two developmental periods. As previously mentioned, most studies rely on GWAS in adult samples and resulting PRSs are tested in child samples, which is problematic given genetic effects can vary with development. We tested the current PRS in a replication sample of children that developmentally aligns with those periods represented in the discovery meta-GWAS. In addition, we tested genetic associations with childhood aggression using the most common measure used in the discovery meta-GWAS (18; parent report of aggression on the Child Behavior Checklist).

Greater alignment of sample characteristics and specificity in the developmental period and phenotypes can help to uncover more precise genetic associations. To address differences in genetic association across development, we considered these associations using a time-varying effect model (TVEM; ^36^) to explicitly model change in the association between PRSs and aggression across childhood.

Based on the discovery meta-GWASs^18^, we created a total of six polygenic risk scores, all at the *p* < 0.05 threshold. For both developmental periods (early and middle childhood), we used meta-GWAS data to create three PRSs: one PRS formed from all SNPs at *p* *<* 0.05, one PRS formed from SNPs that significantly mapped to gene sets using GSEA at *p* *<* 0.05, and finally, one PRS formed from the subset of SNPs that both significantly mapped to gene sets and with biological function at *p* *<* 0.05. We chose *p* *<* 0.05 as a relatively stringent threshold for a number of reasons. First, it includes a smaller number of markers that represent statistically significant associations. Second, by using a more stringent threshold it excludes more chance associations, i.e., SNPs that may be spuriously associated with aggression in the original meta-GWAS. As stated in a recent article, selecting the optimal *p*-value threshold is “analogous to a tuning parameter that balances a signal and noise tradeoff. This tradeoff arises because more significant *p*-value thresholds have higher proportions of causal variants”^37^. This is in-line with our current approach to identify functional variants which is optimized by a more stringent statistical threshold.

To test the utility of these scores, we examined the association of the three early childhood PRSs with aggressive behavior from 2 to 5 years old in a time-varying effect model. We separately tested a similar model in middle childhood in which we examined the association of the three middle childhood PRSs with aggressive behavior from 7.5 to 10.5 years old in a time-varying effect model. The replication sample was drawn from a longitudinal study of child development in which children were randomly assigned to a family-based intervention condition^38^.

We hypothesized that the PRS formed from all SNPs at *p* < 0.05 would not be associated with aggressive behavior given we were not looking to maximize variance explained at multiple thresholds but rather chose a stringent threshold a-priori. Whereas less stringent criteria may explaining greater variance in a phenotype, it also likely includes SNPs that are spuriously associated in the original meta-GWAS or those that have less biological relevance. Further, we hypothesized that the PRS composed of mapped SNPs would show small, albeit significant, associations with aggression and that the PRS composed of functional SNPs would show the most robust associations with child aggression in the early childhood and middle childhood models, respectively. Further, given that PRS were created for unique developmental periods we expected this pattern of findings to be replicated within both the early and middle childhood models, respectively, but there would be no associations between PRSs across developmental periods.

## Online methods

### Participants

Seven hundred and thirty one ethnically and racially diverse, low-income families with 2-year-old children were recruited between 2002 and 2003 from Women, Infants, and Children Nutritional Supplement Programs (WIC) at three sites in Pittsburgh, PA, Eugene, OR, and Charlottesville, VA. Screening procedures were used to recruit families of toddlers at high risk for conduct problems, based on socio-demographic risk, primary caregiver risk, and toddler behavior problems. Participation rates of those families invited to participate who qualified by risk status were high across the three sites [83.2% total (49% female); 84% in Eugene (*n* = 271), 76% in Charlottesville (*n* = 188), 88% in Pittsburgh (*n* = 272)]. More than two thirds of the families reported an annual income of less than $20,000, with 24% of primary caregivers having less than a high school education, 41% having a high school diploma or general education diploma, and an additional 32% having 1–2 years of more than high school education. Primary caregivers (96% mothers) self-identified as belonging to the following ethnic groups: 13% Latino, 28% African American, 50% European American, 13% biracial, and 9% other groups (e.g., Native American, Asian American). For more information about sample characteristics, see ref. ^38^.

Families were randomly assigned to control or intervention conditions after the baseline assessment at child age 2 years, following completion of global ratings by the lead examiner of the assessment. Those in the control condition received WIC services as usual. Those in the intervention condition had the opportunity to receive the Family Check-Up (FCU^39^), following each of the assessments from ages 2 to 10.5. The FCU is comprised of three sessions: (1) assessment, where research staff and parents completed questionnaires about the child’s behavior and family factors, and parents and children were videotaped taking part in structured and unstructured tasks (e.g., free play, clean-up, and teaching tasks in early childhood; discussion tasks with parents during middle childhood); (2) initial interview, where intervention staff and parents discussed their child’s strengths and challenges as well as aspirations the parents had for their child; and (3) feedback, where intervention staff provided feedback to the parents based on the initial interview and assessments. All families were re-contacted at child age 3, 4, 5, 7.5, 8.5, 9.5, and 10.5, and 14 years (81% of the sample participated at age 14) for home-based assessments. In terms of engagement, 76% of families engaged at age 2, with over 90% of the families engaging in at least one session of the FCU by child age 5. Adolescents who were genotyped at age 14 years (86.7% of the sample who participated in home visits at age 14; *n* = 515) comprise the sample for the current study. Selective attrition analyses revealed no significant differences between members of the initial sample with no genetic data and those who were genotyped with respect to parental education, race, gender, study site, child problem behaviors at age 2, temperament, or parental depression.

### Procedures

All assessments were conducted in the home at ages 2–14 with primary caregivers (96% biological mothers at age 2), alternative caregivers when available (e.g., fathers, grandmothers, other relatives involved in the child’s care), and children. Primary caregivers completed questionnaires regarding the physical and socio-cultural environment and children’s behavior. All study protocols were approved by the university Institutional Review Board, parental written consent was obtained for all families (with assent obtained from children beginning at age 14), and families were compensated for their time at each age.

Participants provided saliva samples with Oragene kits for genotyping during the age 14 home visit. RUCDR Infinite Biologics at Rutgers University extracted and normalized the DNA, and then genotyped the samples using the Affymetrix Axiom Biobank1 Array. SNPs that did not meet the criteria of Hardy-Weinberg equilibrium at *p* < 10^−6^ and SNPs with a minor allele frequency less than 1% were removed. Also, any SNP or individual with a missing data rate greater than or equal to 5% was removed (no participants met this criteria). Using the software PLINK, we reduced linkage disequilibrium (LD; correlation among the SNPs) by screening out regions of long-range LD and local LD using the sliding window procedure.

### Measures

#### Polygenic risk scores

Polygenic risk scores were created in three steps. Each step was completed separately for the early childhood meta-GWAS of aggression and the middle childhood meta-GWAS of aggression^18^. The first step involved compiling the reference number and *p*-value for each SNP from the respective meta-GWASs^18^.

Second, SNPs and their respective *p*-values were entered into *I*-GSEA4GWASv2, a web-server that performs GSEA with functional analyses of SNPs^31,32^. This GSEA program uses a two-step method in which each gene is ranked based on the number of SNPs and their respective *p*-values. Genes (and their ranks) are then compared with the available gene sets to calculate each gene sets enrichment score. This software uses a competitive test that compares the proportion of the association between the gene(s) and target gene sets compared with the association between gene(s) outside gene sets^12,13,31,32^. In line-with recommended best practices, the following GSEA specifications were made: SNPs were mapped to genes at 20 kb upstream and downstream from each gene; all gene sets from BioCarta, GO, and KEGG databases were included; and gene set size was restricted to 10–200 genes^12,13,29–32^. At present these options are considered industry standards^12,13^ and ensure that SNPs within or near genes are included and that the most up-to-date gene sets from all possible sources are referenced. Also by including gene sets ranging from 10–200 genes we captured the majority of sizes without being too restrictive or liberal.

Finally, permutation tests are applied at an FDR of *p* < 0.25. For the present analyses, only gene sets with a FDR < 0.05 were retained, which is considered “high confidence” in mapping^30^. SNPs within these gene sets were then extracted at two levels: (a) those that significantly mapped to genes within a gene set, and (b) the subset of those SNPs that were mapped and functional. This procedure was carried out for both meta-GWASs, resulting in mapped and functional SNPs in early and middle childhood.

Finally, PRSs were formed using PLINK^40^. For all PRSs SNPs were coded additively and unit weighted and were formed at the *p* *<* 0.05 threshold. This represents a relatively stringent threshold for SNP inclusion. Whereas less stringent criteria may explain greater variance in a phenotype, it also likely includes SNPs that are spuriously associated in the original meta-GWAS or those that have less biological relevance.

Three PRSs were formed for early childhood and three PRSs were formed for middle childhood. Within each developmental period, PRSs were formed from all SNPs (all-PRS), only those SNPs that significantly mapped to gene sets using GSEA (mapped-PRS), and only the subset of SNPs that both significantly mapped to gene sets and with a known biological function (functional-PRS). All polygenic scores were *Z*-score transformed for interpretability. The number of SNPs within each score can be found in Table 1.

**Table 1:** Correlations, Means and Standard Deviations among Polygenic Risk Scores and Aggression

	Early Ch. All-PRS	Early Ch. Map-PRS	Early Ch. Fun-PRS	Middle Ch. All-PRS	Middle Ch. Map-PRS	Middle Ch. Fun-PRS	Aggression Age 2	Aggression Age 3	Aggression Age 4	Aggression Age 5	Aggression Age 7	Aggression Age 8	Aggression Age 9	Aggression Age 10		
Early Ch. All-PRS (10,571 SNPs)	1															
Early Ch. Map-PRS (184 SNPs)	0.14^**^	1														
Early Ch. Fun-PRS (67 SNPs)	0.13^**^	0.59^***^	1													
Middle Ch. All-PRS (10,612 SNPs)	0.01	0.03	−0.06	1												
Middle Ch. Map-PRS (194 SNPs)	−0.02	0.03	−0.02	0.55^***^	1											
Middle Ch. Fun-PRS (66 SNPs)	−0.01	−0.02	−0.03	.33^***^	0.68^***^	1										
Aggression Age 2	0.01	−0.05	−0.00	−0.05	−0.03	0.01	1									
Aggression Age 3	−0.01	−0.02	0.04	−0.04	0.00	0.02	0.57^***^	1								
Aggression Age 4	0.02	−0.00	.02	−0.01	.01	0.04	0.43^***^	0.62^***^	1							
Aggression Age 5	−0.01	−0.01	0.10*	0.02	0.04	0.07	0.46^***^	0.58^***^	0.61^***^	1						
Aggression Age 7	−0.03	−0.03	0.01	−0.02	0.04	0.05	0.37^***^	0.51^***^	0.57^***^	0.67^***^	1					
Aggression Age 8	0.01	−0.03	0.01	−0.01	−0.01	0.00	0.41^***^	0.49^***^	0.54^***^	0.62^***^	0.73^***^	1				
Aggression Age 9	0.05	−0.02	0.05	0.02	0.02	0.07	0.34^***^	0.48^***^	0.51^***^	0.63^***^	0.73^***^	0.76^***^	1			
Aggression Age 10	0.06	−0.05	−0.00	−0.02	0.02	0.03	0.36^***^	0.42^***^	0.51^***^	0.60^***^	0.65^***^	0.70^***^	0.75^***^	1		
PC1	−0.03	0.05	−0.08	0.76^***^	0.62^***^	0.38^***^	−0.01	−0.02	0.00	0.02	0.05	0.05	0.07	0.04	1	
PC2	0.01	0.02	−0.01	−0.06	0.04	0.02	0.01	0.14^**^	0.11^*^	0.02	0.13^**^	0.08	0.11^*^	0.10^*^	0.00	1
Mean	0.00	−0.01	−0.06	−0.03	−0.10	−0.13	60.13	57.65	56.47	59.33	60.85	58.90	58.79	58.43	0.00	0.00
SD	0.00	0.02	0.03	0.01	0.03	0.04	8.04	7.80	7.76	8.67	10.14	9.65	9.43	9.68	0.04	0.05

Unstandardized PRS are represented in correlation table

*Ch* Childhood, *All-PRS* PRS containing all SNPs, *Map-PRS* PRS containing only mapped SNPs, *Fun-PRS* PRS containing only mapped and functional SNPs, *PC* Ancestry Principal Component

*p < 0.05, **p < 0.01, ***p < 0.001

#### Population admixture

We conducted a Principal Components Analysis of all autosomal SNPs to represent population admixture using PLINK. We extracted the first 20 components, with the first component (PC1) having an eigenvalue of 28.84 and differentiating European-American and Latino groups from African-American groups, with most biracial participants falling in the middle. The second component (PC2) had an eigenvalue of 5.62 and differentiated non-Latino participants (European and African American) from Latino participants. The remaining components had eigenvalues ranging from 1.45 to 1.21 and were excluded from these analyses.

#### CBCL Aggression, 2–10

Primary caregivers completed the Child Behavioral Checklist 1½–5 (CBCL^41^) at the age 2, 3, and 4 visits, and the CBCL 6–18^42^ at the age 5, 7.5, 8.5, 9.5, and 10.5 assessments. Parents rated each item on a 3-point scale (0 = *not true*, 1 = *somewhat or sometimes true*, 2 = *very true or often true*). The aggression subscale was used in the current analyses, which assesses children’s aggressive behavior in early childhood (e.g., “Destroys own things”) and middle childhood (e.g., “Cruel, bullying, or mean to others”). Internal consistency was good across early childhood (αs range 0.85 to 0.90) and middle childhood (αs range 0.91 to 0.92).

#### Covariates

Covariates included gender (females = 0, males = 1; M = 0.51, SD = 0.50), study site location (Eugene and Charlottesville compared to Pittsburgh indexed with two dummy codes), and the first two ancestry principal components, PC1 and PC2. Cumulative risk was also included as a covariate to account for severe environmental influences. This score which was a count-based index created at age 2 composed of seven familial and demographic risk factors. The risk factors included parental substance use, parental education, single adult in the home, overcrowding in the home, parental convictions, neighborhood danger, and poverty. As the study involved an intervention sample, intervention condition was also controlled for in the main analyses.

### Statistical analyses

We examined all relevant statistical assumptions inherent to the application of TVEM (e.g., multivariate normality) and affirmed a priori. All variables were normally distributed^43^ except for PC2, which was square root transformed. TVEM were tested using a time-varying effect model macro in SAS v9.4^44^. TVEMs are an extension of linear regression but make no parametric assumptions about the shape (e.g., linear, quadratic) or rate of change over time in associations^36,44^. Rather, using a regression framework time-varying effect models estimate the shape of change directly from observations by estimating regression coefficients and 95% confidence intervals between time-varying predictors and a longitudinal outcome as a function of continuous time. Significant effects are indicated when the 95% confidence interval around a regression coefficient does not include zero. Therefore, in the current analyses the strength of associations with aggression over time are modeled without any user specification of their shape. At present no formal tests of power exist for TVEM models. However, recent methodological analyses suggest that 100 participants with 10–25 observations per participant is sufficient for reasonable results, and that with more participants fewer observations would be needed^36^. In the current sample we had fewer observations (assessed at ages 2, 3, 4, 5, 7, 8, 9, and 10 years) but greater sample size (*N* = 515). To a degree, power within TVEM models can be observed by the width of the 95% confidence interval, which is dependent on sample size. This can be observed in the current analyses by widening of the interval at the lower and upper ages due to less clustering of observations. Thus, the present findings that occur relative to narrower confidence intervals are likely the most well-powered and reliable.

In the current models we included standardized PRSs as time-varying predictors and covariates as time-invariant effects in-line with TVEM recommendations^36,44^. In the early childhood model we examined the early childhood PRS containing all SNPs at *p* < 0.05, the PRS containing mapped SNPs, and the PRS containing functional SNPs as time-varying predictors of aggression from ages 2–5 years old (actual ages ranged from 24–72 months). Child gender, study site, cumulative risk, intervention condition, and PC1 and PC2 were included as covariates. Within this time-varying effect model, a normal distribution was specified using the P-spline method. A similar time-varying effect model was examined for middle childhood using the age-appropriate PRSs as predictors of aggression from ages 7.5–10.5 (actual ages ranged from 89–142 months), including the same covariates and model settings. Where any significant effects were evident, we subsequently examined the genetic association separately in the intervention and control groups.

## Results

Means, standard deviations, and correlations are presented in Table 1. As expected because of SNP overlap, the early childhood PRSs were significantly correlated with each other. Likewise, the middle childhood PRSs were strongly associated with each other. However, none of the early childhood PRSs were correlated with the middle childhood PRSs. There was only one significant correlation between any PRS and aggression at any age; the functional-PRS was positively associated with age 5 aggression. It should be noted, the middle childhood PRSs were correlated with PC1 (although the functional-PRS had the lowest correlations), which could indicate uncontrolled ancestral variation in the meta-GWAS findings. All analyses removed this variance in the PRS scores by controlling for PC1 and PC2 in all analyses.

### Early childhood

**Fig. 1:**
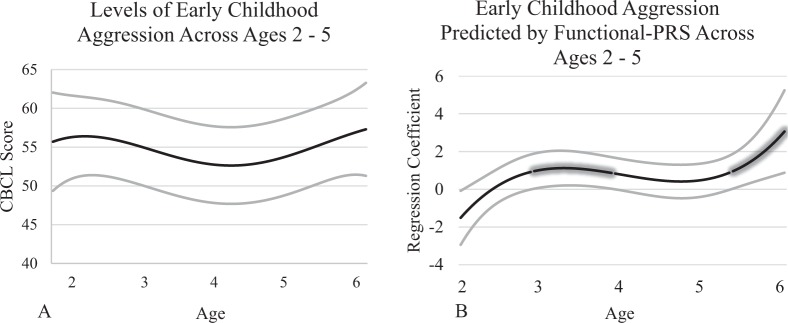
Mean levels of aggression across early childhood (**a**) and associations between the functional polygenic risk score and early childhood aggression (**b**)

Mean levels of aggression across ages 2–5 are presented in Fig. 1a. The all-PRS and mapped-PRS were not significantly associated with aggression at any age. Conversely, the functional-PRS (Fig. 1b) was related to aggression and the change in strength of association from age 2 to 5 years of age was cubic. These findings represent the magnitude of regression coefficients with 95% confidence intervals as a function of age. Significant associations are illustrated by the glowing line which indicates the 95% confidence interval does not include zero. From ∼3 to 4 years, and 5.3 to 6 years, the functional-PRS was significantly associated with aggression. Based on the significant effect of the functional-PRS, we examined the same predictors in separate models for the control group and intervention group. Both groups showed a cubic shape. There were no significant effects in the intervention group, but in the control group a significant association between the functional-PRS and aggression was apparent from ∼5.3 to 6 years. Additionally, as a post-hoc comparison we examined functional-PRS and mapped-PRS at *p* < 0.01 thresholds. The functional-PRS showed a similar association with aggression from ∼3.5 years to 5.75 years, helping to validate the current results.

### Middle childhood

**Fig. 2:**
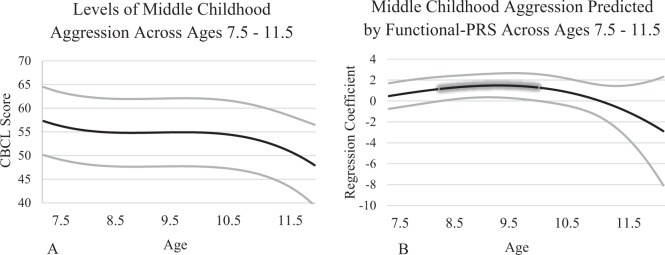
Mean levels of aggression across middle childhood (**a**) and associations between the functional polygenic risk scores and middle childhood aggression (**b**)

Mean levels of aggression across ages 7.5 to 10.5 are presented in Fig. 2a. The all-PRS and mapped-PRS were not significantly associated with aggression at any age. The functional-PRS was associated with aggression and change in the strength of the association had a negative quadratic shape from 7.5 to 10.5 years (Fig. 2b). Significant effects are highlighted by the glowing portions of lines which indicate where the 95% confidence interval does not include zero. From ∼8.75 to 10 years, the functional-PRS was significantly associated with aggression, as indicated by the 95% confidence interval bands. Based on the effect of the functional-PRS, we examined separate models for the control and intervention groups. Both groups presented a mixture of cubic and negative quadratic shapes indicating that results using the full sample reflect both subsamples, but no significant effects were observed in either group. Additionally, as a post-hoc comparison we examined functional-PRS and mapped-PRS at *p* < 0.01 thresholds. The mapped-PRS was associated with aggression at ∼9.0 years old, helping to validate the current results.

## Discussion

To date, the majority of studies in developmental and mental health fields have examined GWAS-based PRSs without considering biological relevance in the selection of SNPs. The present study is the first to create biologically informed PRSs from meta-GWAS data by using GSEA to identify SNPs significantly mapped to gene sets, and those mapped SNPs that were also functional. Broadly, functional-PRSs in both early and middle childhood were associated with aggression, but not the all-PRSs or mapped-PRSs. It should be noted that only one association was observed in zero-order correlations, the functional-PRS was associated with age 5 aggression, which was also reflected in the TVEM model. Additional findings likely reflect the use of time-varying effect models. Time-varying effect models depict associations between the PRSs and behavior over time based on individual time points, whereas correlations do not capture heterogeneity within age or change in behavior.

In addition to this innovative approach, this study also drew SNPs from a meta-GWAS that facilitated creation of separate PRSs for two distinct age groups. Surprisingly, the early and middle childhood PRSs were not associated with each other, indicating largely unique genetic effects on childhood aggression across the two developmental periods. Polygenic risk scores were then examined in relation to aggression in the approximate age ranges reflected in the original meta-GWASs. Further, we examined the CBCL aggression subscale, which was the most commonly used measure in the meta-GWASs. Finally, we utilized time-varying effect models to examine developmental genetic effects on aggression over childhood. Previous developmental genetic theory proposes that genetic effects increase with age because of gene-environment correlations^45,46^, but the current study illustrates that genetic main effects over time may be more nuanced. Results are discussed relative to developmental genetic effects in early and middle childhood.

Previously, a large body of research has identified genetic effects on aggression in early childhood from twin, adoption, and candidate gene research^47–49^. The present findings add a degree of specificity but given the novelty of the current approach should be viewed as preliminary and warrant replication. The functional-PRS in early childhood was associated with aggression from 3 to 4 years old and from ∼5 to 6 years old. Proposedly, the effect of the functional-PRS on aggression from 3 to 4 years may be related to normative trajectories of aggression in early childhood. In particular, aggression increases early in life, peaking from age 2 to 3 which stabilizes but is high from 3 to 4 before declining from 4 to 5 years^50,51^. During this early age children have yet to fully learn to regulate their emotions and behaviors, and temperamental proneness to frustration, negative affect, and low inhibitory control can lead greater anger and aggression in difficult situations^52,53^. These trait-level processes are in part biologically based on developing dopamine, serotonin, and neuroendocrine systems, which may originate from genetic influences^54^. These processes may then be exacerbated by environmental pressures when 3–4 year olds face increasing rules and expectations yet still have limited regulatory capacities. It should be noted that the CBCL aggression subscale assesses other disruptive behaviors besides physical aggression, including anger, frustration, and problems with inhibitory control^41,42^. Thus, the resurgence of the effect of the functional-PRS during 5–6 years of age may also reflect broader disruptive behavior in difficult situations. Biologically based variations in neurotransmitter systems in the face of environmental pressure may contribute to greater levels of aggression^54^. During 5–6 years of age children face numerous transitions including school entry, asserting greater autonomy in parent-child interactions, and spending more time with peers^55^. Seminal research theorizes that individual dispositions are most pronounced during transitions, and relatedly, that genetic effects are accentuated in unstructured situations^56,57^. Thus, the current effects observed during 3–4, and 5–6 may be in part be driven by biological and genetic variation under pressure during developmental transitions when the children begin to have independence (3–4) and during the transition to formal schooling (5–6). Although these patterns of association are theoretically supported they warrant replication.

It is interesting that there was evidence of an association in the control group but not the intervention group. This may indicate that the intervention buffered genetic risk for aggression. However, TVEM does not support tests of significance across models and as testing intervention effectiveness was not the aim of the current study this issue was not pursued. Importantly, this preliminary trend is consistent with previous research findings suggesting that psychosocial intervention effects during early childhood can buffer genetic risk for aggression^58,59^.

As mentioned, these developmental effects may also be explained by variation in biological processes. A list of all gene sets represented by the functional SNPs in early childhood and middle childhood can be found as part of the supplemental materials. For the 67 early childhood functional SNPs, each SNP was represented on average in 5 gene sets, for a total of 354 gene sets, 218 which were unique and 136 which were overlapping. For the 66 middle childhood functional SNPs, each SNP was represented on average in 4 gene sets, for a total of 272 gene sets, 129 of which were unique and 143 which were overlapping. As an illustrative example the SNP rs3744215 was identified in the GSEA within the glutamate receptor activity gene set (GO 0008066, 29) and was subsequently included in the early childhood functional PRS. This SNP is a missense variant located in the glutamate ionotropic receptor NMDA type subunit 2C (GRIN2C) gene and shows elevated presence in the frontal cortex and amygdala^33^. This gene affects glutamate receptor activity related to the excitatory neurotransmitter (NT) glutamate. This NT has been associated with aggression in animal models^60^, but also serves as a metabolic precursor for the inhibitory NT GABA which has also been associated with aggression^61^. Both glutamate receptors^62^ and variants of the GRIN2 gene^63^ have been associated with neurodevelopmental deficits in early childhood. Thus, it may be that early variation in this SNP and gene contribute to variation in glutamate receptor activity, and subsequent aggression in early childhood. While this illustrates a single biological pathway represented by this PRS, many other possibilities were also represented in the PRS such as serotonin and G-protein coupled receptor activity which have been implicated in human and rodent models of aggression^61,64^. This illustrates a potential strength of the present method; facilitating identification of biological pathways. However, it should be noted that even though biologically related SNPs were captured in the current PRS this does not guarantee causal effects on aggression as there are numerous environmental and biological processes that may regulate and modify behavior.

During middle childhood, literature suggests that aggression is fairly stable and slightly decreasing^65,66^. Previous research demonstrates robust and stable genetic effects on aggression during this developmental period^22,67^. In the current study, the functional-PRS was associated with aggression from ∼8.75 to 10.5 years, which bears replication. Hypothetically, these genetic effects may arise from biologically based temperamental predispositions and emerging gene-environment correlations^17,54^. For example, it may be that genetic predispositions for aggression lead to evocative and selection effects, via biological processes, in which more aggressive children both evoke greater aggression (an evocative gene-environment correlation) and affiliate with more aggressive peers (an active evocative gene-environment correlation), which collectively contribute to greater aggression in the child^44^. A complementary explanation is again that during developmental transitions genetic effects may be accentuated^56,57^, and thus during the beginning of middle childhood new social experiences and early puberty may serve to instigate greater expression of individual’s biologically based dispositions. Similarly to early childhood, glutamate and G-protein receptor and signaling pathways were identified via gene sets indicating possible biologically linked processes in child aggression.

Study limitations should also be considered. Results pertain to a high-risk sample so they may not generalize to higher SES populations. In high-risk populations genetic effects may be more pronounced^68,69^. Also, a small proportion of individuals chose not to engage initially or at later waves, despite high retention, which may indicate some evidence of selection bias. However, selective attrition analyses revealed no significant differences between members of the initial sample with no genetic data and those who were genotyped with respect to parental education, race, gender, study site, child problem behaviors at age 2, temperament, or parental depression. An additional limitation is that the primary caregiver (primarily mothers) was the sole reporter for child outcomes, raising the possibility of reporter bias, however parents have shown to be valid reporters of child psychopathology^70^. Finally, genetic associations had small effect sizes on child aggression. This is in line with previous genetics literature using polygenic scores (e.g., 6, 21), and highlights the need for efforts to improve creation of PRSs.

In conclusion, this study illustrates how the current PRSs may be more predictive than previous methods for forming PRSs that include a greater number of SNPs, utilize higher statistical thresholds, and do not filter for biological function. GSEA and other bioinformatics tools can be used to inform selection of genetic variants in forming PRSs to offer more precise tests of genetic influences on behavior. Findings also have important developmental implications for future genetic research that uses GWAS data to form PRSs.

In particular, predictive accuracy may be compromised where sample demographics diverge between the discovery GWAS sample and the sample in which the resulting PRSs will be tested^71^. Based on the present findings and other developmental literature on PRSs, developmentally specific hypotheses should also be made when testing PRSs with attention to the ages represented in the originating GWAS. More optimally, PRSs should be developmentally targeted, with the developmental period of the GWAS matched to the sample in which the PRS is to be tested. In addition, genetic associations should be tested with a phenotypic measure that aligns or is associated with that considered in the original GWAS.

## Supplementary information

Supplemental Tables

## Data Availability

The data that support the findings of this study are available from the corresponding author upon reasonable request and with permission from the study review board.
