# Supplementary material for: Gene set enrichment analysis to create polygenic scores: a developmental examination of aggression
Source: Transl Psychiatry. 2019 Sep 2;9:212. doi: 10.1038/s41398-019-0513-7 (PMC6718657; doi:10.1038/s41398-019-0513-7)
Supplement: Supplementary file 1 — Supplemental Tables [file 41398_2019_513_MOESM1_ESM.docx]

Supplemental Table 1

Early Childhood Functional SNP Gene Sets: For the 67 early childhood functional SNPs, each SNP was represented on average in 5 gene sets. The 67 SNPs were associated with a total of 354 gene sets, 218 which were unique and 136 which were overlapping. GSEA performed using iGSEA4GWASv2 (31, 32).

| **SNP** | **GENE** | **Gene Set Name** | **Gene Set Description** |
| --- | --- | --- | --- |
| rs10512647 | ZNF148 | GO: NEGATIVE REGULATION OF TRANSCRIPTION | Genes annotated by the GO term GO:0016481. Any process that stops, prevents or reduces the frequency, rate or extent of transcription. |
|  |  | GO: NEGATIVE REGULATION OF NUCLEOBASENUCLEOSIDENUCLEOTIDE AND NUCLEIC ACID METABOLIC PROCESS | Genes annotated by the GO term GO:0045934. Any process that stops, prevents or reduces the frequency, rate or extent of the chemical reactions and pathways involving nucleobases, nucleosides, nucleotides and nucleic acids. |
|  |  | GO: NEGATIVE REGULATION OF TRANSCRIPTION FROM RNA POLYMERASE II PROMOTER | Genes annotated by the GO term GO:0000122. Any process that stops, prevents or reduces the frequency, rate or extent of transcription from an RNA polymerase II promoter. |
|  |  | GO: NEGATIVE REGULATION OF TRANSCRIPTION DNA DEPENDENT | Genes annotated by the GO term GO:0045892. Any process that stops, prevents or reduces the frequency, rate or extent of DNA-dependent transcription. |
|  |  | GO: NEGATIVE REGULATION OF RNA METABOLIC PROCESS | Genes annotated by the GO term GO:0051253. Any process that stops, prevents or reduces the frequency, rate or extent of the chemical reactions and pathways involving RNA. |
|  |  | GO: TRANSCRIPTION ACTIVATOR ACTIVITY | Genes annotated by the GO term GO:0016563. Any transcription regulator activity required for initiation or upregulation of transcription. |
|  |  | GO: DNA DIRECTED RNA POLYMERASEII CORE COMPLEX | Genes annotated by the GO term GO:0005665. RNA polymerase II, one of three nuclear DNA-directed RNA polymerases found in all eukaryotes, is a multisubunit complex; typically it produces mRNAs, snoRNAs, and some of the snRNAs. Two large subunits comprise the most conserved portion including the catalytic site and share similarity with other eukaryotic and bacterial multisubunit RNA polymerases. The largest subunit of RNA polymerase II contains an essential carboxyl-terminal domain (CTD) composed of a variable number of heptapeptide repeats (YSPTSPS). The remainder of the complex is composed of smaller subunits (generally ten or more), some of which are also found in RNA polymerases I and III. Although the core is competent to mediate ribonucleic acid synthesis, it requires additional factors to select the appropriate template. |
|  |  | GO: CELLULAR DEFENSE RESPONSE | Genes annotated by the GO term GO:0006968. A defense response that is mediated by cells. |
|  |  |  |  |
| rs1106042 | PIWIL1 | KEGG: DORSO VENTRAL AXIS FORMATION | see KEGG hsa04320 |
|  |  |  |  |
| rs1130409 | APEX1 | GO: TRANSCRIPTION REPRESSOR ACTIVITY | Genes annotated by the GO term GO:0016564. Any transcription regulator activity that prevents or downregulates transcription. |
|  |  | GO: TRANSCRIPTION COREPRESSOR ACTIVITY | Genes annotated by the GO term GO:0003714. The function of a transcription cofactor that represses transcription from a RNA polymerase II promoter; does not bind DNA itself. |
|  |  | GO: DEOXYRIBONUCLEASE ACTIVITY | Genes annotated by the GO term GO:0004536. Catalysis of the hydrolysis of ester linkages within deoxyribonucleic acid. |
|  |  | GO: RESPONSE TO ENDOGENOUS STIMULUS | Genes annotated by the GO term GO:0009719. A change in state or activity of a cell or an organism (in terms of movement, secretion, enzyme production, gene expression, etc.) as a result of an endogenous stimulus. |
|  |  | KEGG: BASE EXCISION REPAIR | see KEGG hsa03410 |
|  |  | GO: DNA REPAIR | Genes annotated by the GO term GO:0006281. The process of restoring DNA after damage. Genomes are subject to damage by chemical and physical agents in the environment (e.g. UV and ionizing radiations, chemical mutagens, fungal and bacterial toxins, etc.) and by free radicals or alkylating agents endogenously generated in metabolism. DNA is also damaged because of errors during its replication. A variety of different DNA repair pathways have been reported that include direct reversal, base excision repair, nucleotide excision repair, photoreactivation, bypass, double-strand break repair pathway, and mismatch repair pathway. |
|  |  | GO: RESPONSE TO DNA DAMAGE STIMULUS | Genes annotated by the GO term GO:0006974. A change in state or activity of a cell or an organism (in terms of movement, secretion, enzyme production, gene expression, etc.) as a result of a stimulus indicating damage to its DNA from environmental insults or errors during metabolism. |
|  |  | GO: ENDODEOXYRIBONUCLEASE ACTIVITY | Genes annotated by the GO term GO:0004520. Catalysis of the hydrolysis of ester linkages within deoxyribonucleic acid by creating internal breaks. |
|  |  | GO: TRANSCRIPTION COACTIVATOR ACTIVITY | Genes annotated by the GO term GO:0003713. The function of a transcription cofactor that activates transcription from a RNA polymerase II promoter; does not bind DNA itself. |
|  |  | GO: TRANSCRIPTION ACTIVATOR ACTIVITY | Genes annotated by the GO term GO:0016563. Any transcription regulator activity required for initiation or upregulation of transcription. |
|  |  | GO: REGULATION OF DNA BINDING | Genes annotated by the GO term GO:0051101. Any process that modulates the frequency, rate or extent of DNA binding, selective interaction with deoxyribonucleic acid. |
|  |  | GO: REGULATION OF BINDING | Genes annotated by the GO term GO:0051098. Any process that modulates the frequency, rate or extent of binding, the selective interaction of a molecule with one or more specific sites on another molecule. |
|  |  |  |  |
| rs1134986 | DLG1 | GO: CELL CELL ADHESION | Genes annotated by the GO term GO:0016337. The attachment of one cell to another cell via adhesion molecules. |
|  |  | GO: INTERCELLULAR JUNCTION | Genes annotated by the GO term GO:0005911. A specialized region of connection between two cells including but not limited to gap junctions, desmosomes, and tight junctions, but excluding direct cytoplasmic junctions such as ring canals. |
|  |  | GO: CYTOSKELETAL PROTEIN BINDING | Genes annotated by the GO term GO:0008092. Interacting selectively with any protein component of any cytoskeleton (actin, microtubule, or intermediate filament cytoskeleton). |
|  |  | GO: PHOSPHOPROTEIN PHOSPHATASE ACTIVITY | Genes annotated by the GO term GO:0004721. Catalysis of the reaction: a phosphoprotein + H2O = a protein + phosphate. Together with protein kinases, these enzymes control the state of phosphorylation of cell proteins and thereby provide an important mechanism for regulating cellular activity. |
|  |  | GO: PHOSPHORIC MONOESTER HYDROLASE ACTIVITY | Genes annotated by the GO term GO:0016791. Catalysis of the hydrolysis of phosphoric monoesters, releasing inorganic phosphate. |
|  |  | KEGG: T CELL RECEPTOR SIGNALING PATHWAY | see KEGG hsa04660 |
|  |  | GO: ENZYME BINDING | Genes annotated by the GO term GO:0019899. Interacting selectively with any enzyme. |
|  |  | GO: ACTIN FILAMENT BASED PROCESS | Genes annotated by the GO term GO:0030029. Any cellular process that depends upon or alters the actin cytoskeleton, that part of the cytoskeleton comprising actin filaments and their associated proteins. |
|  |  |  |  |
| rs11547160 | TCF7L1 | KEGG: ADHERENS JUNCTION | see KEGG hsa04520 |
|  |  | KEGG: ARRHYTHMOGENIC RIGHT VENTRICULAR CARDIOMYOPATHY ARVC | see KEGG hsa05412 |
|  |  | KEGG: MELANOGENESIS | see KEGG hsa04916 |
|  |  | KEGG: WNT SIGNALING PATHWAY | see KEGG hsa04310 |
|  |  |  |  |
| rs11689432 | TARDBP | GO: CYTOSKELETAL PROTEIN BINDING | Genes annotated by the GO term GO:0008092. Interacting selectively with any protein component of any cytoskeleton (actin, microtubule, or intermediate filament cytoskeleton). |
|  |  | GO: M PHASE | Genes annotated by the GO term GO:0000279. Progression through M phase, the part of the cell cycle comprising nuclear division. |
|  |  | GO: MITOSIS | Genes annotated by the GO term GO:0007067. Progression through mitosis, the division of the eukaryotic cell nucleus to produce two daughter nuclei that, usually, contain the identical chromosome complement to their mother. |
|  |  |  |  |
| rs11746232 | LNPEP | GO: PEPTIDASE ACTIVITY | Genes annotated by the GO term GO:0008233. Catalysis of the hydrolysis of peptide bonds. |
|  |  | GO: MULTI ORGANISM PROCESS | Genes annotated by the GO term GO:0051704. The processes by which an organism has an effect on another organism of the same or different species. |
|  |  |  |  |
| rs11852675 | KIF23 | GO: SPINDLE | Genes annotated by the GO term GO:0005819. The array of microtubules and associated molecules that forms between opposite poles of a eukaryotic cell during mitosis or meiosis and serves to move the duplicated chromosomes apart. |
|  |  | GO: KINESIN COMPLEX | Genes annotated by the GO term GO:0005871. Any complex that includes a dimer of molecules from the kinesin superfamily, a group of related proteins that contain an extended region of predicted alpha-helical coiled coil in the main chain that likely produces dimerization. The native complexes of several kinesin family members have also been shown to contain additional peptides, often designated light chains as all of the noncatalytic subunits that are currently known are smaller than the chain that contains the motor unit. Kinesin complexes generally possess a force-generating enzymatic activity, or motor, which converts the free energy of the gamma phosphate bond of ATP into mechanical work. |
|  |  | GO: MICROTUBULE MOTOR ACTIVITY | Genes annotated by the GO term GO:0003777. Catalysis of movement along a microtubule, coupled to the hydrolysis of a nucleoside triphosphate (usually ATP). |
|  |  |  |  |
| rs11888217 | TTN | GO: MUSCLE DEVELOPMENT | Genes annotated by the GO term GO:0007517. The process whose specific outcome is the progression of the muscle over time, from its formation to the mature structure. The muscle is an organ consisting of a tissue made up of various elongated cells that are specialized to contract and thus to produce movement and mechanical work. |
|  |  | GO: SKELETAL MUSCLE DEVELOPMENT | Genes annotated by the GO term GO:0007519. The developmental sequence of events leading to the formation of adult muscle that occurs in the anima. In vertebrate skeletal muscle the main events are: the fusion of myoblasts to form myotubes that increase in size by further fusion to them of myoblasts, the formation of myofibrils within their cytoplasm and the establishment of functional neuromuscular junctions with motor neurons. At this stage they can be regarded as mature muscle fibers. |
|  |  | KEGG: HYPERTROPHIC CARDIOMYOPATHY HCM | see KEGG hsa05410 |
|  |  | GO: STRIATED MUSCLE DEVELOPMENT | Genes annotated by the GO term GO:0014706. The process whose specific outcome is the progression of a striated muscle over time, from its formation to the mature structure. Striated muscle contain fibers that are divided by transverse bands into striations, and cardiac and skeletal muscle are types of striated muscle. Skeletal muscle myoblasts fuse to form myotubes and eventually multinucleated muscle fibers. The fusion of cardiac cells is very rare and can only form binucleate cells. |
|  |  | GO: CONTRACTILE FIBER PART | Genes annotated by the GO term GO:0044449. Any constituent part of a contractile fiber, a fiber composed of actin, myosin, and associated proteins, found in cells of smooth or striated muscle. |
|  |  | KEGG: DILATED CARDIOMYOPATHY | see KEGG hsa05414 |
|  |  | GO: CYTOSKELETAL PROTEIN BINDING | Genes annotated by the GO term GO:0008092. Interacting selectively with any protein component of any cytoskeleton (actin, microtubule, or intermediate filament cytoskeleton). |
|  |  | GO: MYOBLAST DIFFERENTIATION | Genes annotated by the GO term GO:0045445. The process whereby a relatively unspecialized cell acquires specialized features of a myoblast. A myoblast is a mononucleate cell type that, by fusion with other myoblasts, gives rise to the myotubes that eventually develop into skeletal muscle fibers. |
|  |  | GO: MUSCLE CELL DIFFERENTIATION | Genes annotated by the GO term GO:0042692. The process whereby a relatively unspecialized cell acquires specialized features of a muscle cell. |
|  |  | GO: M PHASE | Genes annotated by the GO term GO:0000279. Progression through M phase, the part of the cell cycle comprising nuclear division. |
|  |  | GO: MITOSIS | Genes annotated by the GO term GO:0007067. Progression through mitosis, the division of the eukaryotic cell nucleus to produce two daughter nuclei that, usually, contain the identical chromosome complement to their mother. |
|  |  | GO: CONTRACTILE FIBER | Genes annotated by the GO term GO:0043292. Fibers, composed of actin, myosin, and associated proteins, found in cells of smooth or striated muscle. |
|  |  | GO: ACTIN FILAMENT BASED PROCESS | Genes annotated by the GO term GO:0030029. Any cellular process that depends upon or alters the actin cytoskeleton, that part of the cytoskeleton comprising actin filaments and their associated proteins. |
|  |  |  |  |
| rs12262099 | LRIT1 | GO: INTRINSIC TO ENDOPLASMIC RETICULUM MEMBRANE | Genes annotated by the GO term GO:0031227. Located in the endoplasmic reticulum membrane such that some covalently attached portion of the gene product, for example part of a peptide sequence or some other covalently attached moiety such as a GPI anchor, spans or is embedded in one or both leaflets of the membrane. |
|  |  | GO: INTEGRAL TO ENDOPLASMIC RETICULUM MEMBRANE | Genes annotated by the GO term GO:0030176. Penetrating at least one phospholipid bilayer of an endoplasmic reticulum membrane. May also refer to the state of being buried in the bilayer with no exposure outside the bilayer. |
|  |  |  |  |
| rs12410307 | RAD54L | GO: DNA RECOMBINATION | Genes annotated by the GO term GO:0006310. The processes by which a new genotype is formed by reassortment of genes resulting in gene combinations different from those that were present in the parents. In eukaryotes genetic recombination can occur by chromosome assortment, intrachromosomal recombination, or nonreciprocal interchromosomal recombination. Intrachromosomal recombination occurs by crossing over. In bacteria it may occur by genetic transformation, conjugation, transduction, or F-duction. |
|  |  | GO: RESPONSE TO ENDOGENOUS STIMULUS | Genes annotated by the GO term GO:0009719. A change in state or activity of a cell or an organism (in terms of movement, secretion, enzyme production, gene expression, etc.) as a result of an endogenous stimulus. |
|  |  | GO: DNA REPAIR | Genes annotated by the GO term GO:0006281. The process of restoring DNA after damage. Genomes are subject to damage by chemical and physical agents in the environment (e.g. UV and ionizing radiations, chemical mutagens, fungal and bacterial toxins, etc.) and by free radicals or alkylating agents endogenously generated in metabolism. DNA is also damaged because of errors during its replication. A variety of different DNA repair pathways have been reported that include direct reversal, base excision repair, nucleotide excision repair, photoreactivation, bypass, double-strand break repair pathway, and mismatch repair pathway. |
|  |  | GO: RESPONSE TO DNA DAMAGE STIMULUS | Genes annotated by the GO term GO:0006974. A change in state or activity of a cell or an organism (in terms of movement, secretion, enzyme production, gene expression, etc.) as a result of a stimulus indicating damage to its DNA from environmental insults or errors during metabolism. |
|  |  | GO: MEIOTIC CELL CYCLE | Genes annotated by the GO term GO:0051321. Progression through the phases of the meiotic cell cycle, in which canonically a cell replicates to produce four offspring with half the chromosomal content of the progenitor cell. |
|  |  | KEGG: HOMOLOGOUS RECOMBINATION | see KEGG hsa03440 |
|  |  | GO: M PHASE | Genes annotated by the GO term GO:0000279. Progression through M phase, the part of the cell cycle comprising nuclear division. |
|  |  |  |  |
| rs12598848 | SLC7A5 | GO: AMINO ACID METABOLIC PROCESS | Genes annotated by the GO term GO:0006520. The chemical reactions and pathways involving amino acids, organic acids containing one or more amino substituents. |
|  |  | GO: AMINO ACID AND DERIVATIVE METABOLIC PROCESS | Genes annotated by the GO term GO:0006519. The chemical reactions and pathways involving amino acids, organic acids containing one or more amino substituents, and compounds derived from amino acids. |
|  |  | GO: CARBOXYLIC ACID METABOLIC PROCESS | Genes annotated by the GO term GO:0019752. The chemical reactions and pathways involving carboxylic acids, any organic acid containing one or more carboxyl (COOH) groups or anions (COO-). |
|  |  | GO: ORGANIC ACID METABOLIC PROCESS | Genes annotated by the GO term GO:0006082. The chemical reactions and pathways involving organic acids, any acidic compound containing carbon in covalent linkage. |
|  |  | GO: AMINE TRANSMEMBRANE TRANSPORTER ACTIVITY | Genes annotated by the GO term GO:0005275. Catalysis of the transfer of amines, including polyamines, from one side of the membrane to the other. Amines are organic compounds that are weakly basic in character and contain an amino (-NH2) or substituted amino group. |
|  |  |  |  |
| rs12732063 | MTOR | KEGG: MTOR SIGNALING PATHWAY | see KEGG hsa04150 |
|  |  | BioCarta: BCELLSURVIVAL PATHWAY | B Cell Survival Pathway |
|  |  | KEGG: ADIPOCYTOKINE SIGNALING PATHWAY | see KEGG hsa04920 |
|  |  | KEGG: ERBB SIGNALING PATHWAY | see KEGG hsa04012 |
|  |  |  |  |
| rs12881815 | ESR2 | GO: TRANSCRIPTION COACTIVATOR ACTIVITY | Genes annotated by the GO term GO:0003713. The function of a transcription cofactor that activates transcription from a RNA polymerase II promoter; does not bind DNA itself. |
|  |  | GO: TRANSCRIPTION ACTIVATOR ACTIVITY | Genes annotated by the GO term GO:0016563. Any transcription regulator activity required for initiation or upregulation of transcription. |
|  |  | GO: STEROID HORMONE RECEPTOR SIGNALING PATHWAY | Genes annotated by the GO term GO:0030518. Any series of molecular signals generated as a consequence of a steroid hormone binding to its receptor. |
|  |  |  |  |
| rs1294861 | RAMP3 | KEGG: VASCULAR SMOOTH MUSCLE CONTRACTION | see KEGG hsa04270 |
|  |  | GO: VACUOLE | Genes annotated by the GO term GO:0005773. A closed structure, found only in eukaryotic cells, that is completely surrounded by unit membrane and contains liquid material. Cells contain one or several vacuoles, that may have different functions from each other. Vacuoles have a diverse array of functions. They can act as a storage organelle for nutrients or waste products, as a degradative compartment, as a cost-effective way of increasing cell size, and as a homeostatic regulator controlling both turgor pressure and pH of the cytosol. |
|  |  |  |  |
| rs13020302 | SLC5A7 | GO: SYNAPTIC TRANSMISSION | Genes annotated by the GO term GO:0007268. The process of communication from a neuron to a target (neuron, muscle, or secretory cell) across a synapse. |
|  |  | GO: TRANSMISSION OF NERVE IMPULSE | Genes annotated by the GO term GO:0019226. The sequential electrochemical polarization and depolarization that travels across the membrane of a nerve cell (neuron) in response to stimulation. |
|  |  | GO: NITROGEN COMPOUND BIOSYNTHETIC PROCESS | Genes annotated by the GO term GO:0044271. The chemical reactions and pathways resulting in the formation of organic and inorganic nitrogenous compounds. |
|  |  | GO: SOLUTE SODIUM SYMPORTER ACTIVITY | Genes annotated by the GO term GO:0015370. Catalysis of the transfer of a solute or solutes from one side of a membrane to the other according to the reaction: solute(out) + Na+(out) = solute(in) + Na+(in). |
|  |  | GO: SYMPORTER ACTIVITY | Genes annotated by the GO term GO:0015293. Enables the active transport of a solute across a membrane by a mechanism whereby two or more species are transported together in the same direction in a tightly coupled process not directly linked to a form of energy other than chemiosmotic energy. |
|  |  | GO: AMINE BIOSYNTHETIC PROCESS | Genes annotated by the GO term GO:0009309. The chemical reactions and pathways resulting in the formation of any organic compound that is weakly basic in character and contains an amino or a substituted amino group. Amines are called primary, secondary, or tertiary according to whether one, two, or three carbon atoms are attached to the nitrogen atom. |
|  |  | GO: AMINO ACID AND DERIVATIVE METABOLIC PROCESS | Genes annotated by the GO term GO:0006519. The chemical reactions and pathways involving amino acids, organic acids containing one or more amino substituents, and compounds derived from amino acids. |
|  |  | GO: AMINE TRANSMEMBRANE TRANSPORTER ACTIVITY | Genes annotated by the GO term GO:0005275. Catalysis of the transfer of amines, including polyamines, from one side of the membrane to the other. Amines are organic compounds that are weakly basic in character and contain an amino (-NH2) or substituted amino group. |
|  |  |  |  |
| rs1321311 | CDKN1A | BioCarta: CALCINEURIN PATHWAY | Effects of calcineurin in Keratinocyte Differentiation |
|  |  | BioCarta: ATM PATHWAY | ATM Signaling Pathway |
|  |  | GO: POSITIVE REGULATION OF CELL PROLIFERATION | Genes annotated by the GO term GO:0008284. Any process that activates or increases the rate or extent of cell proliferation. |
|  |  | GO: REGULATION OF PHOSPHORYLATION | Genes annotated by the GO term GO:0042325. Any process that modulates the frequency, rate or extent of addition of phosphate groups into a molecule. |
|  |  | KEGG: ERBB SIGNALING PATHWAY | see KEGG hsa04012 |
|  |  | BioCarta: P53HYPOXIA PATHWAY | Hypoxia and p53 in the Cardiovascular system |
|  |  | GO: NEGATIVE REGULATION OF GROWTH | Genes annotated by the GO term GO:0045926. Any process that stops, prevents or reduces the rate or extent of growth, the increase in size or mass of all or part of an organism. |
|  |  |  |  |
| rs13293384 | TNFSF15 | GO: ACTIVATION OF PROTEIN KINASE ACTIVITY | Genes annotated by the GO term GO:0032147. Any process that initiates the activity of an inactive protein kinase. |
|  |  | GO: REGULATION OF PROTEIN KINASE ACTIVITY | Genes annotated by the GO term GO:0045859. Any process that modulates the frequency, rate or extent of protein kinase activity. |
|  |  | GO: CASPASE ACTIVATION | Genes annotated by the GO term GO:0006919. Upregulation of the activity of a caspase, any of a group of cysteine proteases involved in apoptosis. |
|  |  | GO: REGULATION OF KINASE ACTIVITY | Genes annotated by the GO term GO:0043549. Any process that modulates the frequency, rate or extent of kinase activity, the catalysis of the transfer of a phosphate group, usually from ATP, to a substrate molecule. |
|  |  | GO: REGULATION OF TRANSFERASE ACTIVITY | Genes annotated by the GO term GO:0051338. Any process that modulates the frequency, rate or extent of transferase activity, the catalysis of the transfer of a group, e.g. a methyl group, glycosyl group, acyl group, phosphorus-containing, or other groups, from one compound (generally regarded as the donor) to another compound (generally regarded as the acceptor). Transferase is the systematic name for any enzyme of EC class 2. |
|  |  | GO: POSITIVE REGULATION OF CASPASE ACTIVITY | Genes annotated by the GO term GO:0043280. Any process that activates or increases the activity of a caspase, any of a group of cysteine proteases involved in apoptosis. |
|  |  | GO: POSITIVE REGULATION OF CATALYTIC ACTIVITY | Genes annotated by the GO term GO:0043085. Any process that activates or increases the activity of an enzyme. |
|  |  |  |  |
| rs13898 | AEBP1 | GO: MUSCLE DEVELOPMENT | Genes annotated by the GO term GO:0007517. The process whose specific outcome is the progression of the muscle over time, from its formation to the mature structure. The muscle is an organ consisting of a tissue made up of various elongated cells that are specialized to contract and thus to produce movement and mechanical work. |
|  |  | GO: PEPTIDASE ACTIVITY | Genes annotated by the GO term GO:0008233. Catalysis of the hydrolysis of peptide bonds. |
|  |  | GO: SKELETAL DEVELOPMENT | Genes annotated by the GO term GO:0001501. The process whose specific outcome is the progression of the skeleton over time, from its formation to the mature structure. The skeleton is the bony framework of the body in vertebrates (endoskeleton) or the hard outer envelope of insects (exoskeleton or dermoskeleton). |
|  |  |  |  |
| rs14419 | POLE3 | KEGG: BASE EXCISION REPAIR | see KEGG hsa03410 |
|  |  |  |  |
| rs16850799 | ADIPOR1 | GO: RESPONSE TO ENDOGENOUS STIMULUS | Genes annotated by the GO term GO:0009719. A change in state or activity of a cell or an organism (in terms of movement, secretion, enzyme production, gene expression, etc.) as a result of an endogenous stimulus. |
|  |  | GO: RESPONSE TO HORMONE STIMULUS | Genes annotated by the GO term GO:0009725. A change in state or activity of a cell or an organism (in terms of movement, secretion, enzyme production, gene expression, etc.) as a result of a hormone stimulus. |
|  |  | KEGG: ADIPOCYTOKINE SIGNALING PATHWAY | see KEGG hsa04920 |
|  |  | GO: CARBOXYLIC ACID METABOLIC PROCESS | Genes annotated by the GO term GO:0019752. The chemical reactions and pathways involving carboxylic acids, any organic acid containing one or more carboxyl (COOH) groups or anions (COO-). |
|  |  | GO: ORGANIC ACID METABOLIC PROCESS | Genes annotated by the GO term GO:0006082. The chemical reactions and pathways involving organic acids, any acidic compound containing carbon in covalent linkage. |
|  |  |  |  |
| rs16944971 | FURIN | GO: SERINE TYPE ENDOPEPTIDASE ACTIVITY | Genes annotated by the GO term GO:0004252. Catalysis of the hydrolysis of nonterminal peptide linkages in oligopeptides or polypeptides by a catalytic mechanism that involves a catalytic triad consisting of a serine nucleophile that is activated by a proton relay involving an acidic residue (e.g. aspartate or glutamate) and a basic residue (usually histidine). |
|  |  | GO: TRANS GOLGI NETWORK TRANSPORT VESICLE | Genes annotated by the GO term GO:0030140. A vesicle that mediates transport between the trans-Golgi network and other parts of the cell. |
|  |  | GO: SERINE TYPE PEPTIDASE ACTIVITY | Genes annotated by the GO term GO:0008236. Catalysis of the hydrolysis of peptide linkages in oligopeptides or polypeptides by a catalytic mechanism that involves a catalytic triad consisting of a serine nucleophile that is activated by a proton relay involving an acidic residue (e.g. aspartate or glutamate) and a basic residue (usually histidine). |
|  |  | GO: SERINE HYDROLASE ACTIVITY | Genes annotated by the GO term GO:0017171. Catalysis of the hydrolysis of a substrate by a catalytic mechanism that involves a catalytic triad consisting of a serine nucleophile that is activated by a proton relay involving an acidic residue (e.g. aspartate or glutamate) and a basic residue (usually histidine). |
|  |  | GO: TRANSPORT VESICLE | Genes annotated by the GO term GO:0030133. Any of the vesicles of the constitutive secretory pathway, which carry cargo from the endoplasmic reticulum to the Golgi, between Golgi cisternae, and to destinations within or outside the cell. |
|  |  | GO: PEPTIDASE ACTIVITY | Genes annotated by the GO term GO:0008233. Catalysis of the hydrolysis of peptide bonds. |
|  |  | GO: ENDOPEPTIDASE ACTIVITY | Genes annotated by the GO term GO:0004175. Catalysis of the hydrolysis of nonterminal peptide linkages in oligopeptides or polypeptides, and comprising any enzyme of sub-subclasses EC:3.4.21-99. They are classfied according to the presence of essential catalytic residues or ions at their active sites. |
|  |  | GO: REGULATION OF PROTEIN METABOLIC PROCESS | Genes annotated by the GO term GO:0051246. Any process that modulates the frequency, rate or extent of the chemical reactions and pathways involving a protein. |
|  |  | GO: REGULATION OF CELLULAR PROTEIN METABOLIC PROCESS | Genes annotated by the GO term GO:0032268. Any process that modulates the frequency, rate or extent of the chemical reactions and pathways involving a protein, occurring at the level of an individual cell. |
|  |  |  |  |
| rs17391502 | EXO1 | GO: DEOXYRIBONUCLEASE ACTIVITY | Genes annotated by the GO term GO:0004536. Catalysis of the hydrolysis of ester linkages within deoxyribonucleic acid. |
|  |  | GO: DNA RECOMBINATION | Genes annotated by the GO term GO:0006310. The processes by which a new genotype is formed by reassortment of genes resulting in gene combinations different from those that were present in the parents. In eukaryotes genetic recombination can occur by chromosome assortment, intrachromosomal recombination, or nonreciprocal interchromosomal recombination. Intrachromosomal recombination occurs by crossing over. In bacteria it may occur by genetic transformation, conjugation, transduction, or F-duction. |
|  |  | GO: RESPONSE TO ENDOGENOUS STIMULUS | Genes annotated by the GO term GO:0009719. A change in state or activity of a cell or an organism (in terms of movement, secretion, enzyme production, gene expression, etc.) as a result of an endogenous stimulus. |
|  |  | GO: DNA REPAIR | Genes annotated by the GO term GO:0006281. The process of restoring DNA after damage. Genomes are subject to damage by chemical and physical agents in the environment (e.g. UV and ionizing radiations, chemical mutagens, fungal and bacterial toxins, etc.) and by free radicals or alkylating agents endogenously generated in metabolism. DNA is also damaged because of errors during its replication. A variety of different DNA repair pathways have been reported that include direct reversal, base excision repair, nucleotide excision repair, photoreactivation, bypass, double-strand break repair pathway, and mismatch repair pathway. |
|  |  | GO: RESPONSE TO DNA DAMAGE STIMULUS | Genes annotated by the GO term GO:0006974. A change in state or activity of a cell or an organism (in terms of movement, secretion, enzyme production, gene expression, etc.) as a result of a stimulus indicating damage to its DNA from environmental insults or errors during metabolism. |
|  |  | GO: ENDODEOXYRIBONUCLEASE ACTIVITY | Genes annotated by the GO term GO:0004520. Catalysis of the hydrolysis of ester linkages within deoxyribonucleic acid by creating internal breaks. |
|  |  |  |  |
| rs1799794 | XRCC3 | GO: DNA RECOMBINATION | Genes annotated by the GO term GO:0006310. The processes by which a new genotype is formed by reassortment of genes resulting in gene combinations different from those that were present in the parents. In eukaryotes genetic recombination can occur by chromosome assortment, intrachromosomal recombination, or nonreciprocal interchromosomal recombination. Intrachromosomal recombination occurs by crossing over. In bacteria it may occur by genetic transformation, conjugation, transduction, or F-duction. |
|  |  | GO: RESPONSE TO ENDOGENOUS STIMULUS | Genes annotated by the GO term GO:0009719. A change in state or activity of a cell or an organism (in terms of movement, secretion, enzyme production, gene expression, etc.) as a result of an endogenous stimulus. |
|  |  | GO: DNA REPAIR | Genes annotated by the GO term GO:0006281. The process of restoring DNA after damage. Genomes are subject to damage by chemical and physical agents in the environment (e.g. UV and ionizing radiations, chemical mutagens, fungal and bacterial toxins, etc.) and by free radicals or alkylating agents endogenously generated in metabolism. DNA is also damaged because of errors during its replication. A variety of different DNA repair pathways have been reported that include direct reversal, base excision repair, nucleotide excision repair, photoreactivation, bypass, double-strand break repair pathway, and mismatch repair pathway. |
|  |  | GO: RESPONSE TO DNA DAMAGE STIMULUS | Genes annotated by the GO term GO:0006974. A change in state or activity of a cell or an organism (in terms of movement, secretion, enzyme production, gene expression, etc.) as a result of a stimulus indicating damage to its DNA from environmental insults or errors during metabolism. |
|  |  | KEGG: HOMOLOGOUS RECOMBINATION | see KEGG hsa03440 |
|  |  |  |  |
| rs2044103 | CARD14 | GO: PROTEIN DOMAIN SPECIFIC BINDING | Genes annotated by the GO term GO:0019904. Interacting selectively with a specific domain of a protein. |
|  |  | GO: POSITIVE REGULATION OF PROTEIN METABOLIC PROCESS | Genes annotated by the GO term GO:0051247. Any process that activates or increases the frequency, rate or extent of the chemical reactions and pathways involving a protein. |
|  |  | GO: POSITIVE REGULATION OF CELLULAR PROTEIN METABOLIC PROCESS | Genes annotated by the GO term GO:0032270. Any process that activates or increases the frequency, rate or extent of the chemical reactions and pathways involving a protein, occurring at the level of an individual cell. |
|  |  | GO: POSITIVE REGULATION OF PHOSPHATE METABOLIC PROCESS | Genes annotated by the GO term GO:0045937. Any process that activates or increases the frequency, rate or extent of the chemical reactions and pathways involving phosphates. |
|  |  | GO: POSITIVE REGULATION OF PROTEIN AMINO ACID PHOSPHORYLATION | Genes annotated by the GO term GO:0001934. Any process that activates or increases the frequency, rate or extent of addition of phosphate groups to amino acids within a protein. |
|  |  | GO: POSITIVE REGULATION OF PHOSPHORYLATION | Genes annotated by the GO term GO:0042327. Any process that activates or increases the frequency, rate or extent of addition of phosphate groups to a molecule. |
|  |  | GO: REGULATION OF PROTEIN METABOLIC PROCESS | Genes annotated by the GO term GO:0051246. Any process that modulates the frequency, rate or extent of the chemical reactions and pathways involving a protein. |
|  |  | GO: POSITIVE REGULATION OF PROTEIN MODIFICATION PROCESS | Genes annotated by the GO term GO:0031401. Any process that activates or increases the frequency, rate or extent of the covalent alteration of one or more amino acid residues within a protein. |
|  |  | GO: REGULATION OF PROTEIN MODIFICATION PROCESS | Genes annotated by the GO term GO:0031399. Any process that modulates the frequency, rate or extent of the covalent alteration of one or more amino acid residues within a protein. |
|  |  | GO: REGULATION OF PROTEIN AMINO ACID PHOSPHORYLATION | Genes annotated by the GO term GO:0001932. Any process that modulates the frequency, rate or extent of addition of phosphate groups into an amino acid in a protein. |
|  |  | GO: REGULATION OF CELLULAR PROTEIN METABOLIC PROCESS | Genes annotated by the GO term GO:0032268. Any process that modulates the frequency, rate or extent of the chemical reactions and pathways involving a protein, occurring at the level of an individual cell. |
|  |  | GO: REGULATION OF PHOSPHORYLATION | Genes annotated by the GO term GO:0042325. Any process that modulates the frequency, rate or extent of addition of phosphate groups into a molecule. |
|  |  |  |  |
| rs2104417 | ERGIC3 | GO: ER GOLGI INTERMEDIATE COMPARTMENT | Genes annotated by the GO term GO:0005793. A complex system of membrane-bounded compartments located between endoplasmic reticulum (ER) and the Golgi complex, with a distinctive membrane protein composition; involved in ER-to-Golgi transport. |
|  |  |  |  |
| rs2228058 | PTGER4 | GO: CAMP MEDIATED SIGNALING | Genes annotated by the GO term GO:0019933. A series of molecular signals in which a cell uses cyclic AMP to convert an extracellular signal into a response. |
|  |  | GO: G PROTEIN SIGNALING COUPLED TO CAMP NUCLEOTIDE SECOND MESSENGER | Genes annotated by the GO term GO:0007188. The series of molecular signals generated as a consequence of a G-protein coupled receptor binding to its physiological ligand, followed by modulation of adenylyl cyclase activity and a subsequent change in the concentration of cyclic AMP. |
|  |  |  |  |
| rs2232613 | LBP | GO: INFLAMMATORY RESPONSE | Genes annotated by the GO term GO:0006954. The immediate defensive reaction (by vertebrate tissue) to infection or injury caused by chemical or physical agents. The process is characterized by local vasodilation, extravasation of plasma into intercellular spaces and accumulation of white blood cells and macrophages. |
|  |  | GO: RESPONSE TO WOUNDING | Genes annotated by the GO term GO:0009611. A change in state or activity of a cell or an organism (in terms of movement, secretion, enzyme production, gene expression, etc.) as a result of a stimulus indicating damage to the organism. |
|  |  | GO: CELLULAR DEFENSE RESPONSE | Genes annotated by the GO term GO:0006968. A defense response that is mediated by cells. |
|  |  |  |  |
| rs2234167 | TNFRSF14 | GO: CYTOKINE BINDING | Genes annotated by the GO term GO:0019955. Interacting selectively with a cytokine, any of a group of proteins that function to control the survival, growth and differentiation of tissues and cells, and which have autocrine and paracrine activity. |
|  |  |  |  |
| rs2256480 | CCL27 | GO: G PROTEIN COUPLED RECEPTOR BINDING | Genes annotated by the GO term GO:0001664. Interacting selectively with a G-protein-coupled receptor. |
|  |  |  |  |
| rs2281376 | CROCC | GO: CYTOSKELETAL PROTEIN BINDING | Genes annotated by the GO term GO:0008092. Interacting selectively with any protein component of any cytoskeleton (actin, microtubule, or intermediate filament cytoskeleton). |
|  |  |  |  |
| rs2287694 | GRK6 | GO: REGULATION OF G PROTEIN COUPLED RECEPTOR PROTEIN SIGNALING PATHWAY | Genes annotated by the GO term GO:0008277. Any process that modulates the frequency, rate or extent of G-protein coupled receptor protein signaling pathway activity. |
|  |  |  |  |
| rs2301720 | HOXA7 | BioCarta: KERATINOCYTE PATHWAY | Keratinocyte Differentiation |
|  |  |  |  |
| rs2302234 | PRKAR1A | BioCarta: NFAT PATHWAY | NFAT and Hypertrophy of the heart (Transcription in the broken heart) |
|  |  | BioCarta: BAD PATHWAY | Regulation of BAD phosphorylation |
|  |  | BioCarta: NOS1 PATHWAY | Nitric Oxide Signaling Pathway |
|  |  | KEGG: APOPTOSIS | see KEGG hsa04210 |
|  |  | BioCarta: NO1 PATHWAY | Actions of Nitric Oxide in the Heart |
|  |  | BioCarta: GPCR PATHWAY | Signaling Pathway from G-Protein Families |
|  |  |  |  |
| rs230946 | PMP22 | GO: SYNAPTIC TRANSMISSION | Genes annotated by the GO term GO:0007268. The process of communication from a neuron to a target (neuron, muscle, or secretory cell) across a synapse. |
|  |  | GO: TRANSMISSION OF NERVE IMPULSE | Genes annotated by the GO term GO:0019226. The sequential electrochemical polarization and depolarization that travels across the membrane of a nerve cell (neuron) in response to stimulation. |
|  |  | GO: PERIPHERAL NERVOUS SYSTEM DEVELOPMENT | Genes annotated by the GO term GO:0007422. The process whose specific outcome is the progression of the peripheral nervous system over time, from its formation to the mature structure. The peripheral nervous system is one of the two major divisions of the nervous system. Nerves in the PNS connect the central nervous system (CNS) with sensory organs, other organs, muscles, blood vessels and glands. |
|  |  |  |  |
| rs2445857 | ZNF274 | GO: TRANSCRIPTION REPRESSOR ACTIVITY | Genes annotated by the GO term GO:0016564. Any transcription regulator activity that prevents or downregulates transcription. |
|  |  | GO: TRANSCRIPTION COREPRESSOR ACTIVITY | Genes annotated by the GO term GO:0003714. The function of a transcription cofactor that represses transcription from a RNA polymerase II promoter; does not bind DNA itself. |
|  |  | KEGG: NEUROTROPHIN SIGNALING PATHWAY | see KEGG hsa04722 |
|  |  |  |  |
| rs27036 | ERAP1 | GO: PEPTIDASE ACTIVITY | Genes annotated by the GO term GO:0008233. Catalysis of the hydrolysis of peptide bonds. |
|  |  | GO: ANATOMICAL STRUCTURE FORMATION | Genes annotated by the GO term GO:0048646. The process pertaining to the initial formation of an anatomical structure from unspecified parts. This process begins with the specific processes that contribute to the appearance of the discrete structure and ends when the structural rudiment is recognizable. An anatomical structure is any biological entity that occupies space and is distinguished from its surroundings. Anatomical structures can be macroscopic such as a carpel, or microscopic such as an acrosome. |
|  |  | GO: GROWTH FACTOR ACTIVITY | Genes annotated by the GO term GO:0008083. The function that stimulates a cell to grow or proliferate. Most growth factors have other actions besides the induction of cell growth or proliferation. |
|  |  | GO: ANGIOGENESIS | Genes annotated by the GO term GO:0001525. Blood vessel formation when new vessels emerge from the proliferation of pre-existing blood vessels. |
|  |  | GO: VASCULATURE DEVELOPMENT | Genes annotated by the GO term GO:0001944. The process whose specific outcome is the progression of the vasculature over time, from its formation to the mature structure. |
|  |  | GO: ORGAN MORPHOGENESIS | Genes annotated by the GO term GO:0009887. Morphogenesis of an organ. An organ is defined as a tissue or set of tissues that work together to perform a specific function or functions. Morphogenesis is the process by which anatomical structures are generated and organized. Organs are commonly observed as visibly distinct structures, but may also exist as loosely associated clusters of cells that work together to perform a specific function or functions. |
|  |  |  |  |
| rs3730089 | PIK3R1 | BioCarta: NKCELLS PATHWAY | Ras-Independent pathway in NK cell-mediated cytotoxicity |
|  |  | BioCarta: GCR PATHWAY | Corticosteroids and cardioprotection |
|  |  | BioCarta: AKT PATHWAY | AKT Signaling Pathway |
|  |  | BioCarta: FCER1 PATHWAY | Fc Epsilon Receptor I Signaling in Mast Cells |
|  |  | BioCarta: TCR PATHWAY | T Cell Receptor Signaling Pathway |
|  |  | BioCarta: HDAC PATHWAY | Control of skeletal myogenesis by HDAC and calcium/calmodulin-dependent kinase (CaMK) |
|  |  | KEGG: FC GAMMA R MEDIATED PHAGOCYTOSIS | see KEGG hsa04666 |
|  |  | BioCarta: GH PATHWAY | Growth Hormone Signaling Pathway |
|  |  | KEGG: MTOR SIGNALING PATHWAY | see KEGG hsa04150 |
|  |  | BioCarta: TPO PATHWAY | TPO Signaling Pathway |
|  |  | KEGG: NATURAL KILLER CELL MEDIATED CYTOTOXICITY | see KEGG hsa04650 |
|  |  | BioCarta: NFAT PATHWAY | NFAT and Hypertrophy of the heart (Transcription in the broken heart) |
|  |  | KEGG: JAK STAT SIGNALING PATHWAY | see KEGG hsa04630 |
|  |  | BioCarta: BAD PATHWAY | Regulation of BAD phosphorylation |
|  |  | BioCarta: BCELLSURVIVAL PATHWAY | B Cell Survival Pathway |
|  |  | BioCarta: EGF PATHWAY | EGF Signaling Pathway |
|  |  | BioCarta: NGF PATHWAY | Nerve growth factor pathway (NGF) |
|  |  | BioCarta: MET PATHWAY | Signaling of Hepatocyte Growth Factor Receptor |
|  |  | KEGG: NEUROTROPHIN SIGNALING PATHWAY | see KEGG hsa04722 |
|  |  | BioCarta: CXCR4 PATHWAY | CXCR4 Signaling Pathway |
|  |  | BioCarta: HER2 PATHWAY | Role of ERBB2 in Signal Transduction and Oncology |
|  |  | BioCarta: IGF1 PATHWAY | IGF-1 Signaling Pathway |
|  |  | KEGG: FC EPSILON RI SIGNALING PATHWAY | see KEGG hsa04664 |
|  |  | KEGG: APOPTOSIS | see KEGG hsa04210 |
|  |  | BioCarta: PDGF PATHWAY | PDGF Signaling Pathway |
|  |  | GO: ENZYME LINKED RECEPTOR PROTEIN SIGNALING PATHWAY | Genes annotated by the GO term GO:0007167. Any series of molecular signals initiated by the binding of an extracellular ligand to a receptor on the surface of the target cell, where the receptor possesses catalytic activity or is closely associated with an enzyme such as a protein kinase. |
|  |  | BioCarta: IL7 PATHWAY | IL-7 Signal Transduction |
|  |  | BioCarta: GLEEVEC PATHWAY | Inhibition of Cellular Proliferation by Gleevec |
|  |  | BioCarta: ACH PATHWAY | Role of nicotinic acetylcholine receptors in the regulation of apoptosis |
|  |  | BioCarta: RAS PATHWAY | Ras Signaling Pathway |
|  |  | GO: TRANSMEMBRANE RECEPTOR PROTEIN TYROSINE KINASE SIGNALING PATHWAY | Genes annotated by the GO term GO:0007169. The series of molecular signals generated as a consequence of a transmembrane receptor tyrosine kinase binding to its physiological ligand. |
|  |  | KEGG: T CELL RECEPTOR SIGNALING PATHWAY | see KEGG hsa04660 |
|  |  | KEGG: ALDOSTERONE REGULATED SODIUM REABSORPTION | see KEGG hsa04960 |
|  |  | GO: CYTOSOLIC PART | Genes annotated by the GO term GO:0044445. Any constituent part of cytosol, that part of the cytoplasm that does not contain membranous or particulate subcellular components. |
|  |  | BioCarta: CDC42RAC PATHWAY | Role of PI3K subunit p85 in regulation of Actin Organization and Cell Migration |
|  |  | GO: ENZYME BINDING | Genes annotated by the GO term GO:0019899. Interacting selectively with any enzyme. |
|  |  | BioCarta: TRKA PATHWAY | Trka Receptor Signaling Pathway |
|  |  | KEGG: ERBB SIGNALING PATHWAY | see KEGG hsa04012 |
|  |  | KEGG: VEGF SIGNALING PATHWAY | see KEGG hsa04370 |
|  |  | BioCarta: PTEN PATHWAY | PTEN dependent cell cycle arrest and apoptosis |
|  |  | BioCarta: IL2RB PATHWAY | IL-2 Receptor Beta Chain in T cell Activation |
|  |  | KEGG: PHOSPHATIDYLINOSITOL SIGNALING SYSTEM | see KEGG hsa04070 |
|  |  |  |  |
| rs3739142 | REG1A | GO: POSITIVE REGULATION OF CELL PROLIFERATION | Genes annotated by the GO term GO:0008284. Any process that activates or increases the rate or extent of cell proliferation. |
|  |  |  |  |
| rs3744215 | GRIN2C | GO: GLUTAMATE SIGNALING PATHWAY | Genes annotated by the GO term GO:0007215. The series of molecular signals generated as a consequence of glutamate binding to a cell surface receptor. |
|  |  | BioCarta: NOS1 PATHWAY | Nitric Oxide Signaling Pathway |
|  |  | GO: GLUTAMATE RECEPTOR ACTIVITY | Genes annotated by the GO term GO:0008066. Combining with glutamate to initiate a change in cell activity. |
|  |  | KEGG: LONG TERM POTENTIATION | see KEGG hsa04720 |
|  |  |  |  |
| rs3745902 | KIR3DL1 | KEGG: NATURAL KILLER CELL MEDIATED CYTOTOXICITY | see KEGG hsa04650 |
|  |  |  |  |
| rs3763046 | NRTN | GO: NEURON DIFFERENTIATION | Genes annotated by the GO term GO:0030182. The process whereby a relatively unspecialized cell acquires specialized features of a neuron. |
|  |  | GO: CELL MIGRATION | Genes annotated by the GO term GO:0016477. The orderly movement of cells from one site to another, often during the development of a multicellular organism. |
|  |  | GO: GENERATION OF NEURONS | Genes annotated by the GO term GO:0048699. The process by which nerve cells are generated. This includes the production of neuroblasts and their differentiation into neurons. |
|  |  | GO: NEURON DEVELOPMENT | Genes annotated by the GO term GO:0048666. The process whose specific outcome is the progression of a neuron over time, from initial commitment of the cell to a specific fate, to the fully functional differentiated cell. |
|  |  | GO: NEURITE DEVELOPMENT | Genes annotated by the GO term GO:0031175. The process whose specific outcome is the progression of the neurite over time, from its formation to the mature structure. The neurite is any process extending from a neural cell, such as axons or dendrites. |
|  |  | GO: NEUROGENESIS | Genes annotated by the GO term GO:0022008. Generation of cells within the nervous system. |
|  |  | GO: ENZYME LINKED RECEPTOR PROTEIN SIGNALING PATHWAY | Genes annotated by the GO term GO:0007167. Any series of molecular signals initiated by the binding of an extracellular ligand to a receptor on the surface of the target cell, where the receptor possesses catalytic activity or is closely associated with an enzyme such as a protein kinase. |
|  |  | GO: TRANSMEMBRANE RECEPTOR PROTEIN TYROSINE KINASE SIGNALING PATHWAY | Genes annotated by the GO term GO:0007169. The series of molecular signals generated as a consequence of a transmembrane receptor tyrosine kinase binding to its physiological ligand. |
|  |  | GO: MAPKKK CASCADE GO 0000165 | Genes annotated by the GO term GO:0000165. Cascade of at least three protein kinase activities culminating in the phosphorylation and activation of a MAP kinase. MAPKKK cascades lie downstream of numerous signaling pathways. |
|  |  |  |  |
| rs3802264 | ZHX2 | GO: NEGATIVE REGULATION OF TRANSCRIPTION | Genes annotated by the GO term GO:0016481. Any process that stops, prevents or reduces the frequency, rate or extent of transcription. |
|  |  | GO: NEGATIVE REGULATION OF NUCLEOBASENUCLEOSIDENUCLEOTIDE AND NUCLEIC ACID METABOLIC PROCESS | Genes annotated by the GO term GO:0045934. Any process that stops, prevents or reduces the frequency, rate or extent of the chemical reactions and pathways involving nucleobases, nucleosides, nucleotides and nucleic acids. |
|  |  | GO: NEGATIVE REGULATION OF TRANSCRIPTION DNA DEPENDENT | Genes annotated by the GO term GO:0045892. Any process that stops, prevents or reduces the frequency, rate or extent of DNA-dependent transcription. |
|  |  | GO: NEGATIVE REGULATION OF RNA METABOLIC PROCESS | Genes annotated by the GO term GO:0051253. Any process that stops, prevents or reduces the frequency, rate or extent of the chemical reactions and pathways involving RNA. |
|  |  |  |  |
| rs3817198 | LSP1 | GO: ACTIN BINDING | Genes annotated by the GO term GO:0003779. Interacting selectively with monomeric or multimeric forms of actin, including actin filaments. |
|  |  | GO: ACTIN CYTOSKELETON | Genes annotated by the GO term GO:0015629. The part of the cytoskeleton (the internal framework of a cell) composed of actin and associated proteins. Includes actin cytoskeleton-associated complexes. |
|  |  | GO: CYTOSKELETAL PROTEIN BINDING | Genes annotated by the GO term GO:0008092. Interacting selectively with any protein component of any cytoskeleton (actin, microtubule, or intermediate filament cytoskeleton). |
|  |  | GO: CELLULAR DEFENSE RESPONSE | Genes annotated by the GO term GO:0006968. A defense response that is mediated by cells. |
|  |  |  |  |
| rs3819331 | PTS | GO: AMINO ACID METABOLIC PROCESS | Genes annotated by the GO term GO:0006520. The chemical reactions and pathways involving amino acids, organic acids containing one or more amino substituents. |
|  |  | GO: AMINO ACID AND DERIVATIVE METABOLIC PROCESS | Genes annotated by the GO term GO:0006519. The chemical reactions and pathways involving amino acids, organic acids containing one or more amino substituents, and compounds derived from amino acids. |
|  |  | GO: CARBOXYLIC ACID METABOLIC PROCESS | Genes annotated by the GO term GO:0019752. The chemical reactions and pathways involving carboxylic acids, any organic acid containing one or more carboxyl (COOH) groups or anions (COO-). |
|  |  | GO: ORGANIC ACID METABOLIC PROCESS | Genes annotated by the GO term GO:0006082. The chemical reactions and pathways involving organic acids, any acidic compound containing carbon in covalent linkage. |
|  |  | GO: CENTRAL NERVOUS SYSTEM DEVELOPMENT | Genes annotated by the GO term GO:0007417. The process whose specific outcome is the progression of the central nervous system over time, from its formation to the mature structure. The central nervous system is the core nervous system that serves an integrating and coordinating function. In vertebrates it consists of the brain, spinal cord and spinal nerves. In those invertebrates with a central nervous system it typically consists of a brain, cerebral ganglia and a nerve cord. |
|  |  |  |  |
| rs3862476 | QPRT | GO: SYNAPTIC TRANSMISSION | Genes annotated by the GO term GO:0007268. The process of communication from a neuron to a target (neuron, muscle, or secretory cell) across a synapse. |
|  |  | GO: TRANSFERASE ACTIVITY TRANSFERRING GLYCOSYL GROUPS | Genes annotated by the GO term GO:0016757. Catalysis of the transfer of a glycosyl group from one compound (donor) to another (acceptor). |
|  |  | GO: TRANSMISSION OF NERVE IMPULSE | Genes annotated by the GO term GO:0019226. The sequential electrochemical polarization and depolarization that travels across the membrane of a nerve cell (neuron) in response to stimulation. |
|  |  | GO: GENERATION OF PRECURSOR METABOLITES AND ENERGY | Genes annotated by the GO term GO:0006091. The chemical reactions and pathways resulting in the formation of precursor metabolites, substances from which energy is derived, and the processes involved in the liberation of energy from these substances. |
|  |  | KEGG: NICOTINATE AND NICOTINAMIDE METABOLISM | see KEGG hsa00760 |
|  |  |  |  |
| rs4149570 | TNFRSF1A | GO: INFLAMMATORY RESPONSE | Genes annotated by the GO term GO:0006954. The immediate defensive reaction (by vertebrate tissue) to infection or injury caused by chemical or physical agents. The process is characterized by local vasodilation, extravasation of plasma into intercellular spaces and accumulation of white blood cells and macrophages. |
|  |  | GO: CYTOKINE BINDING | Genes annotated by the GO term GO:0019955. Interacting selectively with a cytokine, any of a group of proteins that function to control the survival, growth and differentiation of tissues and cells, and which have autocrine and paracrine activity. |
|  |  | GO: RESPONSE TO WOUNDING | Genes annotated by the GO term GO:0009611. A change in state or activity of a cell or an organism (in terms of movement, secretion, enzyme production, gene expression, etc.) as a result of a stimulus indicating damage to the organism. |
|  |  | GO: REGULATION OF RESPONSE TO EXTERNAL STIMULUS | Genes annotated by the GO term GO:0032101. Any process that modulates the frequency, rate or extent of a response to an external stimulus. |
|  |  | BioCarta: PML PATHWAY | Regulation of transcriptional activity by PML |
|  |  | GO: REGULATION OF RESPONSE TO STIMULUS | Genes annotated by the GO term GO:0048583. Any process that modulates the frequency, rate or extent of a response to a stimulus. Response to stimulus is a change in state or activity of a cell or an organism (in terms of movement, secretion, enzyme production, gene expression, etc.) as a result of a stimulus. |
|  |  | GO: POSITIVE REGULATION OF RESPONSE TO STIMULUS | Genes annotated by the GO term GO:0048584. Any process that activates, maintains or increases the rate of a response to a stimulus. Response to stimulus is a change in state or activity of a cell or an organism (in terms of movement, secretion, enzyme production, gene expression, etc.) as a result of a stimulus. |
|  |  | GO: POSITIVE REGULATION OF TRANSCRIPTION | Genes annotated by the GO term GO:0045941. Any process that activates or increases the frequency, rate or extent of transcription. |
|  |  | BioCarta: KERATINOCYTE PATHWAY | Keratinocyte Differentiation |
|  |  | KEGG: APOPTOSIS | see KEGG hsa04210 |
|  |  | GO: POSITIVE REGULATION OF NUCLEOBASENUCLEOSIDENUCLEOTIDE AND NUCLEIC ACID METABOLIC PROCESS | Genes annotated by the GO term GO:0045935. Any process that activates or increases the frequency, rate or extent of the chemical reactions and pathways involving nucleobases, nucleosides, nucleotides and nucleic acids. |
|  |  | KEGG: ADIPOCYTOKINE SIGNALING PATHWAY | see KEGG hsa04920 |
|  |  | GO: CARBOXYLIC ACID METABOLIC PROCESS | Genes annotated by the GO term GO:0019752. The chemical reactions and pathways involving carboxylic acids, any organic acid containing one or more carboxyl (COOH) groups or anions (COO-). |
|  |  | GO: ORGANIC ACID METABOLIC PROCESS | Genes annotated by the GO term GO:0006082. The chemical reactions and pathways involving organic acids, any acidic compound containing carbon in covalent linkage. |
|  |  | GO: POSITIVE REGULATION OF TRANSCRIPTIONDNA DEPENDENT | Genes annotated by the GO term GO:0045893. Any process that activates or increases the frequency, rate or extent of DNA-dependent transcription. |
|  |  | BioCarta: NFKB PATHWAY | NF-kB Signaling Pathway |
|  |  | GO: REGULATION OF DEFENSE RESPONSE | Genes annotated by the GO term GO:0031347. Any process that modulates the frequency, rate or extent of a defense response. |
|  |  | GO: POSITIVE REGULATION OF TRANSCRIPTION FROM RNA POLYMERASE II PROMOTER | Genes annotated by the GO term GO:0045944. Any process that activates or increases the frequency, rate or extent of transcription from the RNA polymerase II promoter. |
|  |  | BioCarta: CERAMIDE PATHWAY | Ceramide Signaling Pathway |
|  |  | GO: POSITIVE REGULATION OF RNA METABOLIC PROCESS | Genes annotated by the GO term GO:0051254. Any process that activates or increases the frequency, rate or extent of the chemical reactions and pathways involving RNA. |
|  |  |  |  |
| rs41506651 | ERAP2 | GO: PEPTIDASE ACTIVITY | Genes annotated by the GO term GO:0008233. Catalysis of the hydrolysis of peptide bonds. |
|  |  |  |  |
| rs422674 | CABIN1 | BioCarta: HDAC PATHWAY | Control of skeletal myogenesis by HDAC and calcium/calmodulin-dependent kinase (CaMK) |
|  |  | BioCarta: MEF2D PATHWAY | Role of MEF2D in T-cell Apoptosis |
|  |  |  |  |
| rs4253399 | F11 | GO: SERINE TYPE ENDOPEPTIDASE ACTIVITY | Genes annotated by the GO term GO:0004252. Catalysis of the hydrolysis of nonterminal peptide linkages in oligopeptides or polypeptides by a catalytic mechanism that involves a catalytic triad consisting of a serine nucleophile that is activated by a proton relay involving an acidic residue (e.g. aspartate or glutamate) and a basic residue (usually histidine). |
|  |  | GO: SERINE TYPE PEPTIDASE ACTIVITY | Genes annotated by the GO term GO:0008236. Catalysis of the hydrolysis of peptide linkages in oligopeptides or polypeptides by a catalytic mechanism that involves a catalytic triad consisting of a serine nucleophile that is activated by a proton relay involving an acidic residue (e.g. aspartate or glutamate) and a basic residue (usually histidine). |
|  |  | GO: SERINE HYDROLASE ACTIVITY | Genes annotated by the GO term GO:0017171. Catalysis of the hydrolysis of a substrate by a catalytic mechanism that involves a catalytic triad consisting of a serine nucleophile that is activated by a proton relay involving an acidic residue (e.g. aspartate or glutamate) and a basic residue (usually histidine). |
|  |  | GO: PEPTIDASE ACTIVITY | Genes annotated by the GO term GO:0008233. Catalysis of the hydrolysis of peptide bonds. |
|  |  | GO: ENDOPEPTIDASE ACTIVITY | Genes annotated by the GO term GO:0004175. Catalysis of the hydrolysis of nonterminal peptide linkages in oligopeptides or polypeptides, and comprising any enzyme of sub-subclasses EC:3.4.21-99. They are classfied according to the presence of essential catalytic residues or ions at their active sites. |
|  |  | KEGG: COMPLEMENT AND COAGULATION CASCADES | see KEGG hsa04610 |
|  |  |  |  |
| rs4440299 | WFS1 | GO: GENERATION OF PRECURSOR METABOLITES AND ENERGY | Genes annotated by the GO term GO:0006091. The chemical reactions and pathways resulting in the formation of precursor metabolites, substances from which energy is derived, and the processes involved in the liberation of energy from these substances. |
|  |  | GO: SENSORY PERCEPTION | Genes annotated by the GO term GO:0007600. The series of events required for an organism to receive a sensory stimulus, convert it to a molecular signal, and recognize and characterize the signal. |
|  |  |  |  |
| rs4646049 | CASP9 | BioCarta: AKT PATHWAY | AKT Signaling Pathway |
|  |  | GO: PEPTIDASE ACTIVITY | Genes annotated by the GO term GO:0008233. Catalysis of the hydrolysis of peptide bonds. |
|  |  | GO: ENDOPEPTIDASE ACTIVITY | Genes annotated by the GO term GO:0004175. Catalysis of the hydrolysis of nonterminal peptide linkages in oligopeptides or polypeptides, and comprising any enzyme of sub-subclasses EC:3.4.21-99. They are classfied according to the presence of essential catalytic residues or ions at their active sites. |
|  |  | KEGG: APOPTOSIS | see KEGG hsa04210 |
|  |  | GO: CASPASE ACTIVATION | Genes annotated by the GO term GO:0006919. Upregulation of the activity of a caspase, any of a group of cysteine proteases involved in apoptosis. |
|  |  | BioCarta: RAS PATHWAY | Ras Signaling Pathway |
|  |  | KEGG: VEGF SIGNALING PATHWAY | see KEGG hsa04370 |
|  |  | BioCarta: MITOCHONDRIA PATHWAY | Role of Mitochondria in Apoptotic Signaling |
|  |  | BioCarta: HSP27 PATHWAY | Stress Induction of HSP Regulation |
|  |  | GO: POSITIVE REGULATION OF CASPASE ACTIVITY | Genes annotated by the GO term GO:0043280. Any process that activates or increases the activity of a caspase, any of a group of cysteine proteases involved in apoptosis. |
|  |  | GO: POSITIVE REGULATION OF CATALYTIC ACTIVITY | Genes annotated by the GO term GO:0043085. Any process that activates or increases the activity of an enzyme. |
|  |  |  |  |
| rs4770684 | PARP4 | GO: INFLAMMATORY RESPONSE | Genes annotated by the GO term GO:0006954. The immediate defensive reaction (by vertebrate tissue) to infection or injury caused by chemical or physical agents. The process is characterized by local vasodilation, extravasation of plasma into intercellular spaces and accumulation of white blood cells and macrophages. |
|  |  | GO: RESPONSE TO WOUNDING | Genes annotated by the GO term GO:0009611. A change in state or activity of a cell or an organism (in terms of movement, secretion, enzyme production, gene expression, etc.) as a result of a stimulus indicating damage to the organism. |
|  |  | KEGG: BASE EXCISION REPAIR | see KEGG hsa03410 |
|  |  | GO: ENZYME BINDING | Genes annotated by the GO term GO:0019899. Interacting selectively with any enzyme. |
|  |  |  |  |
| rs4781680 | MYH11 | GO: MUSCLE DEVELOPMENT | Genes annotated by the GO term GO:0007517. The process whose specific outcome is the progression of the muscle over time, from its formation to the mature structure. The muscle is an organ consisting of a tissue made up of various elongated cells that are specialized to contract and thus to produce movement and mechanical work. |
|  |  | GO: SMOOTH MUSCLE CONTRACTION GO 0006939 | Genes annotated by the GO term GO:0006939. A process whereby force is generated within smooth muscle tissue, resulting in a change in muscle geometry. Force generation involves a chemo-mechanical energy conversion step. The chemo-mechanical energy conversion step is carried out by the actin/myosin complex activity, which generates force through ATP hydrolysis. Smooth muscle differs from striated muscle in the much higher actin/myosin ratio, the absence of conspicuous sarcomeres and the ability to contract to a much smaller fraction of its resting length. |
|  |  | GO: SKELETAL MUSCLE DEVELOPMENT | Genes annotated by the GO term GO:0007519. The developmental sequence of events leading to the formation of adult muscle that occurs in the anima. In vertebrate skeletal muscle the main events are: the fusion of myoblasts to form myotubes that increase in size by further fusion to them of myoblasts, the formation of myofibrils within their cytoplasm and the establishment of functional neuromuscular junctions with motor neurons. At this stage they can be regarded as mature muscle fibers. |
|  |  | KEGG: VASCULAR SMOOTH MUSCLE CONTRACTION | see KEGG hsa04270 |
|  |  | GO: STRIATED MUSCLE DEVELOPMENT | Genes annotated by the GO term GO:0014706. The process whose specific outcome is the progression of a striated muscle over time, from its formation to the mature structure. Striated muscle contain fibers that are divided by transverse bands into striations, and cardiac and skeletal muscle are types of striated muscle. Skeletal muscle myoblasts fuse to form myotubes and eventually multinucleated muscle fibers. The fusion of cardiac cells is very rare and can only form binucleate cells. |
|  |  | KEGG: TIGHT JUNCTION | see KEGG hsa04530 |
|  |  | GO: MYOBLAST DIFFERENTIATION | Genes annotated by the GO term GO:0045445. The process whereby a relatively unspecialized cell acquires specialized features of a myoblast. A myoblast is a mononucleate cell type that, by fusion with other myoblasts, gives rise to the myotubes that eventually develop into skeletal muscle fibers. |
|  |  | GO: MUSCLE CELL DIFFERENTIATION | Genes annotated by the GO term GO:0042692. The process whereby a relatively unspecialized cell acquires specialized features of a muscle cell. |
|  |  | GO: ACTIN FILAMENT BASED PROCESS | Genes annotated by the GO term GO:0030029. Any cellular process that depends upon or alters the actin cytoskeleton, that part of the cytoskeleton comprising actin filaments and their associated proteins. |
|  |  |  |  |
| rs4984636 | CACNA1H | GO: MUSCLE DEVELOPMENT | Genes annotated by the GO term GO:0007517. The process whose specific outcome is the progression of the muscle over time, from its formation to the mature structure. The muscle is an organ consisting of a tissue made up of various elongated cells that are specialized to contract and thus to produce movement and mechanical work. |
|  |  | GO: SKELETAL MUSCLE DEVELOPMENT | Genes annotated by the GO term GO:0007519. The developmental sequence of events leading to the formation of adult muscle that occurs in the anima. In vertebrate skeletal muscle the main events are: the fusion of myoblasts to form myotubes that increase in size by further fusion to them of myoblasts, the formation of myofibrils within their cytoplasm and the establishment of functional neuromuscular junctions with motor neurons. At this stage they can be regarded as mature muscle fibers. |
|  |  | GO: REGULATION OF HEART CONTRACTION | Genes annotated by the GO term GO:0008016. Any process that modulates the frequency, rate or extent of heart contraction. Heart contraction is the process by which the heart decreases in volume in a characteristic way to propel blood through the body. |
|  |  | GO: REGULATION OF MULTICELLULAR ORGANISMAL PROCESS | Genes annotated by the GO term GO:0051239. Any process that modulates the frequency, rate or extent of an organismal process, the processes pertinent to the function of an organism above the cellular level; includes the integrated processes of tissues and organs. |
|  |  | GO: STRIATED MUSCLE DEVELOPMENT | Genes annotated by the GO term GO:0014706. The process whose specific outcome is the progression of a striated muscle over time, from its formation to the mature structure. Striated muscle contain fibers that are divided by transverse bands into striations, and cardiac and skeletal muscle are types of striated muscle. Skeletal muscle myoblasts fuse to form myotubes and eventually multinucleated muscle fibers. The fusion of cardiac cells is very rare and can only form binucleate cells. |
|  |  | GO: SUBSTRATE SPECIFIC CHANNEL ACTIVITY | Genes annotated by the GO term GO:0022838. Catalysis of energy-independent facilitated diffusion, mediated by passage of a specific solute through a transmembrane aqueous pore or channel. Stereospecificity is not exhibited but this transport may be specific for a particular molecular species or class of molecules. |
|  |  | GO: ION CHANNEL ACTIVITY | Genes annotated by the GO term GO:0005216. Catalysis of facilitated diffusion of an ion (by an energy-independent process) by passage through a transmembrane aqueous pore or channel without evidence for a carrier-mediated mechanism. |
|  |  | GO: VOLTAGE GATED CALCIUM CHANNEL ACTIVITY | Genes annotated by the GO term GO:0005245. Catalysis of the transmembrane transfer of a calcium ion by a voltage-gated channel. |
|  |  | GO: MYOBLAST DIFFERENTIATION | Genes annotated by the GO term GO:0045445. The process whereby a relatively unspecialized cell acquires specialized features of a myoblast. A myoblast is a mononucleate cell type that, by fusion with other myoblasts, gives rise to the myotubes that eventually develop into skeletal muscle fibers. |
|  |  | GO: METAL ION TRANSMEMBRANE TRANSPORTER ACTIVITY | Genes annotated by the GO term GO:0046873. Catalysis of the transfer of metal ions from one side of a membrane to the other. |
|  |  | GO: MUSCLE CELL DIFFERENTIATION | Genes annotated by the GO term GO:0042692. The process whereby a relatively unspecialized cell acquires specialized features of a muscle cell. |
|  |  | GO: CALCIUM CHANNEL ACTIVITY | Genes annotated by the GO term GO:0005262. Catalysis of facilitated diffusion of an calcium (by an energy-independent process) involving passage through a transmembrane aqueous pore or channel without evidence for a carrier-mediated mechanism. |
|  |  | GO: GATED CHANNEL ACTIVITY | Genes annotated by the GO term GO:0022836. Catalysis of the transmembrane transfer of a solute by a channel that opens in response to a specific stimulus. |
|  |  |  |  |
| rs4987082 | PHB | GO: HISTONE MODIFICATION | Genes annotated by the GO term GO:0016570. The covalent alteration of one or more amino acid residues within a histone protein. |
|  |  | GO: TRANSCRIPTION REPRESSOR ACTIVITY | Genes annotated by the GO term GO:0016564. Any transcription regulator activity that prevents or downregulates transcription. |
|  |  | GO: NEGATIVE REGULATION OF TRANSCRIPTION | Genes annotated by the GO term GO:0016481. Any process that stops, prevents or reduces the frequency, rate or extent of transcription. |
|  |  | GO: COVALENT CHROMATIN MODIFICATION | Genes annotated by the GO term GO:0016569. The alteration of DNA or protein in chromatin by the covalent addition or removal of chemical groups. |
|  |  | GO: NEGATIVE REGULATION OF NUCLEOBASENUCLEOSIDENUCLEOTIDE AND NUCLEIC ACID METABOLIC PROCESS | Genes annotated by the GO term GO:0045934. Any process that stops, prevents or reduces the frequency, rate or extent of the chemical reactions and pathways involving nucleobases, nucleosides, nucleotides and nucleic acids. |
|  |  | GO: CHROMATIN MODIFICATION | Genes annotated by the GO term GO:0016568. The alteration of DNA or protein in chromatin, which may result in changing the chromatin structure. |
|  |  | GO: CHROMOSOME ORGANIZATION AND BIOGENESIS | Genes annotated by the GO term GO:0051276. A process that is carried out at the cellular level that results in the formation, arrangement of constituent parts, or disassembly of chromosomes, structures composed of a very long molecule of DNA and associated proteins that carries hereditary information. |
|  |  | GO: MITOCHONDRIAL INNER MEMBRANE | Genes annotated by the GO term GO:0005743. The inner, i.e. lumen-facing, lipid bilayer of the mitochondrial envelope. It is highly folded to form cristae. |
|  |  | GO: TRANSCRIPTION ACTIVATOR ACTIVITY | Genes annotated by the GO term GO:0016563. Any transcription regulator activity required for initiation or upregulation of transcription. |
|  |  | GO: ESTABLISHMENT AND OR MAINTENANCE OF CHROMATIN ARCHITECTURE | Genes annotated by the GO term GO:0006325. The specification, formation and maintenance of the physical structure of eukaryotic chromatin. |
|  |  | GO: ORGANELLE INNER MEMBRANE | Genes annotated by the GO term GO:0019866. The inner, i.e. lumen-facing, lipid bilayer of an organelle envelope; usually highly selective to most ions and metabolites. |
|  |  |  |  |
| rs6058522 | HCK | KEGG: FC GAMMA R MEDIATED PHAGOCYTOSIS | see KEGG hsa04666 |
|  |  | GO: MESODERM DEVELOPMENT | Genes annotated by the GO term GO:0007498. The process whose specific outcome is the progression of the mesoderm over time, from its formation to the mature structure. The mesoderm is the middle germ layer that develops into muscle, bone, cartilage, blood and connective tissue. |
|  |  | BioCarta: BARRESTIN SRC PATHWAY | Roles of fl-arrestin-dependent Recruitment of Src Kinases in GPCR Signaling |
|  |  |  |  |
| rs720793 | ZMPSTE24 | GO: PEPTIDASE ACTIVITY | Genes annotated by the GO term GO:0008233. Catalysis of the hydrolysis of peptide bonds. |
|  |  |  |  |
| rs7254474 | CDC37 | GO: REGULATION OF PROTEIN KINASE ACTIVITY | Genes annotated by the GO term GO:0045859. Any process that modulates the frequency, rate or extent of protein kinase activity. |
|  |  | GO: REGULATION OF CYCLIN DEPENDENT PROTEIN KINASE ACTIVITY | Genes annotated by the GO term GO:0000079. Any process that modulates the frequency, rate or extent of CDK activity. |
|  |  | GO: REGULATION OF KINASE ACTIVITY | Genes annotated by the GO term GO:0043549. Any process that modulates the frequency, rate or extent of kinase activity, the catalysis of the transfer of a phosphate group, usually from ATP, to a substrate molecule. |
|  |  | GO: REGULATION OF TRANSFERASE ACTIVITY | Genes annotated by the GO term GO:0051338. Any process that modulates the frequency, rate or extent of transferase activity, the catalysis of the transfer of a group, e.g. a methyl group, glycosyl group, acyl group, phosphorus-containing, or other groups, from one compound (generally regarded as the donor) to another compound (generally regarded as the acceptor). Transferase is the systematic name for any enzyme of EC class 2. |
|  |  |  |  |
| rs7633618 | CPA3 | GO: PEPTIDASE ACTIVITY | Genes annotated by the GO term GO:0008233. Catalysis of the hydrolysis of peptide bonds. |
|  |  |  |  |
| rs7859021 | FXN | GO: SYNAPTIC TRANSMISSION | Genes annotated by the GO term GO:0007268. The process of communication from a neuron to a target (neuron, muscle, or secretory cell) across a synapse. |
|  |  | GO: DI TRI VALENT INORGANIC CATION TRANSMEMBRANE TRANSPORTER ACTIVITY | Genes annotated by the GO term GO:0015082. Catalysis of the transfer of inorganic cations with a valency of two or three from one side of the membrane to the other. Inorganic cations are atoms or small molecules with a positive charge that do not contain carbon in covalent linkage. |
|  |  | GO: TRANSMISSION OF NERVE IMPULSE | Genes annotated by the GO term GO:0019226. The sequential electrochemical polarization and depolarization that travels across the membrane of a nerve cell (neuron) in response to stimulation. |
|  |  | GO: GENERATION OF PRECURSOR METABOLITES AND ENERGY | Genes annotated by the GO term GO:0006091. The chemical reactions and pathways resulting in the formation of precursor metabolites, substances from which energy is derived, and the processes involved in the liberation of energy from these substances. |
|  |  | GO: INORGANIC CATION TRANSMEMBRANE TRANSPORTER ACTIVITY | Genes annotated by the GO term GO:0022890. Catalysis of the transfer of inorganic cations from one side of a membrane to the other. Inorganic cations are atoms or small molecules with a positive charge that do not contain carbon in covalent linkage. |
|  |  | GO: METAL ION TRANSMEMBRANE TRANSPORTER ACTIVITY | Genes annotated by the GO term GO:0046873. Catalysis of the transfer of metal ions from one side of a membrane to the other. |
|  |  | GO: ELECTRON TRANSPORT GO 0006118 | Genes annotated by the GO term GO:0006118. The transport of electrons from an electron donor to an electron acceptor. |
|  |  | GO: CELLULAR HOMEOSTASIS | Genes annotated by the GO term GO:0019725. The processes involved in the maintenance of an internal equilibrium at the level of the cell. |
|  |  |  |  |
| rs7943716 | CAPN5 | GO: PEPTIDASE ACTIVITY | Genes annotated by the GO term GO:0008233. Catalysis of the hydrolysis of peptide bonds. |
|  |  | GO: ENDOPEPTIDASE ACTIVITY | Genes annotated by the GO term GO:0004175. Catalysis of the hydrolysis of nonterminal peptide linkages in oligopeptides or polypeptides, and comprising any enzyme of sub-subclasses EC:3.4.21-99. They are classfied according to the presence of essential catalytic residues or ions at their active sites. |
|  |  |  |  |
| rs8065080 | TRPV1 | GO: SUBSTRATE SPECIFIC CHANNEL ACTIVITY | Genes annotated by the GO term GO:0022838. Catalysis of energy-independent facilitated diffusion, mediated by passage of a specific solute through a transmembrane aqueous pore or channel. Stereospecificity is not exhibited but this transport may be specific for a particular molecular species or class of molecules. |
|  |  | GO: ION CHANNEL ACTIVITY | Genes annotated by the GO term GO:0005216. Catalysis of facilitated diffusion of an ion (by an energy-independent process) by passage through a transmembrane aqueous pore or channel without evidence for a carrier-mediated mechanism. |
|  |  | GO: METAL ION TRANSMEMBRANE TRANSPORTER ACTIVITY | Genes annotated by the GO term GO:0046873. Catalysis of the transfer of metal ions from one side of a membrane to the other. |
|  |  | GO: CALCIUM CHANNEL ACTIVITY | Genes annotated by the GO term GO:0005262. Catalysis of facilitated diffusion of an calcium (by an energy-independent process) involving passage through a transmembrane aqueous pore or channel without evidence for a carrier-mediated mechanism. |
|  |  |  |  |
| rs8102919 | CASP14 | GO: PEPTIDASE ACTIVITY | Genes annotated by the GO term GO:0008233. Catalysis of the hydrolysis of peptide bonds. |
|  |  | GO: ENDOPEPTIDASE ACTIVITY | Genes annotated by the GO term GO:0004175. Catalysis of the hydrolysis of nonterminal peptide linkages in oligopeptides or polypeptides, and comprising any enzyme of sub-subclasses EC:3.4.21-99. They are classfied according to the presence of essential catalytic residues or ions at their active sites. |
|  |  |  |  |
| rs910873 | PIGU | GO: JAK STAT CASCADE | Genes annotated by the GO term GO:0007259. The processes by which STAT proteins (Signal Transducers and Activators of Transcription) are activated by members of the JAK (janus activated kinase) family of tyrosine kinases, following the binding of cytokines to their cognate receptor. Once activated, STATs dimerize and translocate to the nucleus and modulate the expression of target genes. |
|  |  | GO: INTRINSIC TO ENDOPLASMIC RETICULUM MEMBRANE | Genes annotated by the GO term GO:0031227. Located in the endoplasmic reticulum membrane such that some covalently attached portion of the gene product, for example part of a peptide sequence or some other covalently attached moiety such as a GPI anchor, spans or is embedded in one or both leaflets of the membrane. |
|  |  | GO: INTEGRAL TO ENDOPLASMIC RETICULUM MEMBRANE | Genes annotated by the GO term GO:0030176. Penetrating at least one phospholipid bilayer of an endoplasmic reticulum membrane. May also refer to the state of being buried in the bilayer with no exposure outside the bilayer. |
|  |  | GO: PROTEIN AMINO ACID LIPIDATION | Genes annotated by the GO term GO:0006497. The covalent or non-covalent attachment of lipid moieties to an amino acid in a protein. |
|  |  |  |  |
| rs9509307 | IFT88 | GO: EXCRETION | Genes annotated by the GO term GO:0007588. The elimination by an organism of the waste products that arise as a result of metabolic activity. These products include water, carbon dioxide (CO2), and nitrogenous compounds. |
|  |  |  |  |
| rs953062 | SLC25A27 | GO: GENERATION OF PRECURSOR METABOLITES AND ENERGY | Genes annotated by the GO term GO:0006091. The chemical reactions and pathways resulting in the formation of precursor metabolites, substances from which energy is derived, and the processes involved in the liberation of energy from these substances. |

Supplemental Table 2

Middle Childhood Functional SNP Gene Sets: For the 66 middle childhood functional SNPs, each SNP was represented on average in 4 gene sets. The 66 SNPs were associated with a total of 272 gene sets, 129 of which were unique and 143 which were overlapping.

| **SNP** | **GENE** | **Gene Set Name** | **Gene Set Description** |
| --- | --- | --- | --- |
| rs10235371 | FIGNL1 | GO: NUCLEOTIDE METABOLIC PROCESS | Genes annotated by the GO term GO:0009117. The chemical reactions and pathways involving a nucleotide, a nucleoside that is esterified with (ortho)phosphate or an oligophosphate at any hydroxyl group on the glycose moiety; may be mono-, di- or triphosphate; this definition includes cyclic-nucleotides (nucleoside cyclic phosphates). |
|  |  |  |  |
| rs10260248 | TAS2R40 | KEGG: TASTE TRANSDUCTION | see KEGG hsa04742 |
|  |  |  |  |
| rs10281008 | TAX1BP1 | GO: NEGATIVE REGULATION OF DEVELOPMENTAL PROCESS | Genes annotated by the GO term GO:0051093. Any process that stops, prevents or reduces the rate or extent of development, the biological process whose specific outcome is the progression of an organism over time from an initial condition (e.g. a zygote, or a young adult) to a later condition (e.g. a multicellular animal or an aged adult). |
|  |  | GO: NEGATIVE REGULATION OF PROGRAMMED CELL DEATH | Genes annotated by the GO term GO:0043069. Any process that stops, prevents or reduces the frequency, rate or extent of programmed cell death, cell death resulting from activation of endogenous cellular processes. |
|  |  | GO: NEGATIVE REGULATION OF APOPTOSIS | Genes annotated by the GO term GO:0043066. Any process that stops, prevents or reduces the frequency, rate or extent of cell death by apoptosis. |
|  |  | GO: ANTI APOPTOSIS | Genes annotated by the GO term GO:0006916. A process which directly inhibits any of the steps required for cell death by apoptosis. |
|  |  |  |  |
| rs10419393 | CYP2A7 | KEGG: RETINOL METABOLISM | see KEGG hsa00830 |
|  |  | KEGG: DRUG METABOLISM OTHER ENZYMES | see KEGG hsa00983 |
|  |  |  |  |
| rs10431392 | TBX3 | GO: EMBRYONIC MORPHOGENESIS | Genes annotated by the GO term GO:0048598. The process by which anatomical structures are generated and organized during the embryonic phase. Morphogenesis pertains to the creation of form. The embryonic phase begins with zygote formation. The end of the embryonic phase is organism-specific. For example, it would be at birth for mammals, larval hatching for insects and seed dormancy in plants. |
|  |  | GO: SKELETAL DEVELOPMENT | Genes annotated by the GO term GO:0001501. The process whose specific outcome is the progression of the skeleton over time, from its formation to the mature structure. The skeleton is the bony framework of the body in vertebrates (endoskeleton) or the hard outer envelope of insects (exoskeleton or dermoskeleton). |
|  |  | GO: NEGATIVE REGULATION OF DEVELOPMENTAL PROCESS | Genes annotated by the GO term GO:0051093. Any process that stops, prevents or reduces the rate or extent of development, the biological process whose specific outcome is the progression of an organism over time from an initial condition (e.g. a zygote, or a young adult) to a later condition (e.g. a multicellular animal or an aged adult). |
|  |  | GO: GLAND DEVELOPMENT | Genes annotated by the GO term GO:0048732. The process whose specific outcome is the progression of a gland over time, from its formation to the mature structure. A gland is an organ specialised for secretion. |
|  |  | GO: PATTERN SPECIFICATION PROCESS | Genes annotated by the GO term GO:0007389. The developmental processes that result in the creation of defined areas or spaces within an organism to which cells respond and eventually are instructed to differentiate. |
|  |  | GO: AGING | Genes annotated by the GO term GO:0007568. The inherent decline over time, from the optimal fertility and viability of early maturity, that may precede death and may be preceded by other indications, such as sterility. |
|  |  | GO: GENERATION OF A SIGNAL INVOLVED IN CELL CELL SIGNALING | Genes annotated by the GO term GO:0003001. The cellular process by which a physical entity or change in state, a signal, is created that originates in one cell and is used to transfer information to another cell. This process begins with the initial formation of the signal and ends with the mature form and placement of the signal. |
|  |  | GO: NEGATIVE REGULATION OF PROGRAMMED CELL DEATH | Genes annotated by the GO term GO:0043069. Any process that stops, prevents or reduces the frequency, rate or extent of programmed cell death, cell death resulting from activation of endogenous cellular processes. |
|  |  | GO: NEGATIVE REGULATION OF TRANSCRIPTION DNA DEPENDENT | Genes annotated by the GO term GO:0045892. Any process that stops, prevents or reduces the frequency, rate or extent of DNA-dependent transcription. |
|  |  | GO: NEGATIVE REGULATION OF APOPTOSIS | Genes annotated by the GO term GO:0043066. Any process that stops, prevents or reduces the frequency, rate or extent of cell death by apoptosis. |
|  |  | GO: ORGAN MORPHOGENESIS | Genes annotated by the GO term GO:0009887. Morphogenesis of an organ. An organ is defined as a tissue or set of tissues that work together to perform a specific function or functions. Morphogenesis is the process by which anatomical structures are generated and organized. Organs are commonly observed as visibly distinct structures, but may also exist as loosely associated clusters of cells that work together to perform a specific function or functions. |
|  |  | GO: ANTI APOPTOSIS | Genes annotated by the GO term GO:0006916. A process which directly inhibits any of the steps required for cell death by apoptosis. |
|  |  | GO: EMBRYONIC DEVELOPMENT | Genes annotated by the GO term GO:0009790. The process whose specific outcome is the progression of an embryo from its formation until the end of its embryonic life stage. The end of the embryonic stage is organism-specific. For example, for mammals, the process would begin with zygote formation and end with birth. For insects, the process would begin at zygote formation and end with larval hatching. For plant zygotic embryos, this would be from zygote formation to the end of seed dormancy. For plant vegetative embryos, this would be from the initial determination of the cell or group of cells to form an embryo until the point when the embryo becomes independent of the parent plant. |
|  |  | GO: NEGATIVE REGULATION OF TRANSCRIPTION | Genes annotated by the GO term GO:0016481. Any process that stops, prevents or reduces the frequency, rate or extent of transcription. |
|  |  | GO: RNA POLYMERASE II TRANSCRIPTION FACTOR ACTIVITY | Genes annotated by the GO term GO:0003702. Functions to initiate or regulate RNA polymerase II transcription. |
|  |  | GO: HORMONE SECRETION | Genes annotated by the GO term GO:0046879. The regulated release of hormones, substances with a specific regulatory effect on a particular organ or group of cells. |
|  |  | GO: NEGATIVE REGULATION OF RNA METABOLIC PROCESS | Genes annotated by the GO term GO:0051253. Any process that stops, prevents or reduces the frequency, rate or extent of the chemical reactions and pathways involving RNA. |
|  |  | GO: TRANSCRIPTION REPRESSOR ACTIVITY | Genes annotated by the GO term GO:0016564. Any transcription regulator activity that prevents or downregulates transcription. |
|  |  | GO: REGIONALIZATION | Genes annotated by the GO term GO:0003002. The pattern specification process by which an axis or axes is subdivided in space to define an area or volume in which specific patterns of cell differentiation will take place or in which cells interpret a specific environment. |
|  |  | GO: NEGATIVE REGULATION OF CELL DIFFERENTIATION | Genes annotated by the GO term GO:0045596. Any process that stops, prevents or reduces the frequency, rate or extent of cell differentiation. |
|  |  | GO: REGULATION OF CELL DIFFERENTIATION | Genes annotated by the GO term GO:0045595. Any process that modulates the frequency, rate or extent of cell differentiation, the process whereby relatively unspecialized cells acquire specialized structural and functional features. |
|  |  | GO: SECRETION | Genes annotated by the GO term GO:0046903. The regulated release of a substance by a cell, a group of cells, or a tissue. |
|  |  |  |  |
| rs1051858 | ARID4A | GO: NEGATIVE REGULATION OF TRANSCRIPTION DNA DEPENDENT | Genes annotated by the GO term GO:0045892. Any process that stops, prevents or reduces the frequency, rate or extent of DNA-dependent transcription. |
|  |  | GO: NEGATIVE REGULATION OF TRANSCRIPTION | Genes annotated by the GO term GO:0016481. Any process that stops, prevents or reduces the frequency, rate or extent of transcription. |
|  |  | GO: NEGATIVE REGULATION OF RNA METABOLIC PROCESS | Genes annotated by the GO term GO:0051253. Any process that stops, prevents or reduces the frequency, rate or extent of the chemical reactions and pathways involving RNA. |
|  |  | GO: TRANSCRIPTION REPRESSOR ACTIVITY | Genes annotated by the GO term GO:0016564. Any transcription regulator activity that prevents or downregulates transcription. |
|  |  |  |  |
| rs1052748 | PLD2 | GO: PHOSPHORIC ESTER HYDROLASE ACTIVITY | Genes annotated by the GO term GO:0042578. Catalysis of the reaction: RPO-R' + H2O = RPOOH + R'H. This reaction is the hydrolysis of any phosphoric ester bond, any ester formed from orthophosphoric acid, O=P(OH)3. |
|  |  | GO: PHOSPHORIC DIESTER HYDROLASE ACTIVITY | Genes annotated by the GO term GO:0008081. Catalysis of the hydrolysis of a phosphodiester to give a phosphomonoester and a free hydroxyl group. |
|  |  | KEGG: FC GAMMA R MEDIATED PHAGOCYTOSIS | see KEGG hsa04666 |
|  |  |  |  |
| rs10893295 | ROBO3 | KEGG: AXON GUIDANCE | see KEGG hsa04360 |
|  |  |  |  |
| rs11080149 | NF1 | GO: SECOND MESSENGER MEDIATED SIGNALING | Genes annotated by the GO term GO:0019932. A series of molecular signals in which an ion or small molecule is formed or released into the cytosol, thereby helping relay the signal within the cell. |
|  |  | GO: G PROTEIN SIGNALING COUPLED TO CYCLIC NUCLEOTIDE SECOND MESSENGER | Genes annotated by the GO term GO:0007187. The series of molecular signals generated as a consequence of a G-protein coupled receptor binding to its physiological ligand, followed by modulation of a nucleotide cyclase activity and a subsequent change in the concentration of a cyclic nucleotide. |
|  |  | GO: SKELETAL DEVELOPMENT | Genes annotated by the GO term GO:0001501. The process whose specific outcome is the progression of the skeleton over time, from its formation to the mature structure. The skeleton is the bony framework of the body in vertebrates (endoskeleton) or the hard outer envelope of insects (exoskeleton or dermoskeleton). |
|  |  | GO: BONE REMODELING | Genes annotated by the GO term GO:0046849. The continuous turnover of bone matrix and mineral that involves first, an increase in resorption (osteoclastic activity) and later, reactive bone formation (osteoblastic activity). The process of bone remodeling takes place in the adult skeleton at discrete foci. The process ensures the mechanical integrity of the skeleton throughout life and plays an important role in calcium homeostasis. An imbalance in the regulation of bone resorption and bone formation results in many of the metabolic bone diseases, such as osteoporosis. |
|  |  | GO: CYCLIC NUCLEOTIDE MEDIATED SIGNALING | Genes annotated by the GO term GO:0019935. A series of molecular signals in which a cell uses a cyclic nucleotide to convert an extracellular signal into a response. |
|  |  | GO: CAMP MEDIATED SIGNALING | Genes annotated by the GO term GO:0019933. A series of molecular signals in which a cell uses cyclic AMP to convert an extracellular signal into a response. |
|  |  | GO: G PROTEIN SIGNALING COUPLED TO CAMP NUCLEOTIDE SECOND MESSENGER | Genes annotated by the GO term GO:0007188. The series of molecular signals generated as a consequence of a G-protein coupled receptor binding to its physiological ligand, followed by modulation of adenylyl cyclase activity and a subsequent change in the concentration of cyclic AMP. |
|  |  | GO: TISSUE REMODELING | Genes annotated by the GO term GO:0048771. The reorganization or renovation of existing tissues. This process can either change the characteristics of a tissue such as in blood vessel remodeling, or result in the dynamic equilibrium of a tissue such as in bone remodeling. |
|  |  | GO: NEGATIVE REGULATION OF DEVELOPMENTAL PROCESS | Genes annotated by the GO term GO:0051093. Any process that stops, prevents or reduces the rate or extent of development, the biological process whose specific outcome is the progression of an organism over time from an initial condition (e.g. a zygote, or a young adult) to a later condition (e.g. a multicellular animal or an aged adult). |
|  |  | GO: GLAND DEVELOPMENT | Genes annotated by the GO term GO:0048732. The process whose specific outcome is the progression of a gland over time, from its formation to the mature structure. A gland is an organ specialised for secretion. |
|  |  | GO: ENDOTHELIAL CELL PROLIFERATION | Genes annotated by the GO term GO:0001935. The multiplication or reproduction of endothelial cells, resulting in the expansion of a cell population. Endothelial cells are thin flattened cells which line the inside surfaces of body cavities, blood vessels, and lymph vessels, making up the endothelium. |
|  |  | GO: PERIPHERAL NERVOUS SYSTEM DEVELOPMENT | Genes annotated by the GO term GO:0007422. The process whose specific outcome is the progression of the peripheral nervous system over time, from its formation to the mature structure. The peripheral nervous system is one of the two major divisions of the nervous system. Nerves in the PNS connect the central nervous system (CNS) with sensory organs, other organs, muscles, blood vessels and glands. |
|  |  | GO: ANGIOGENESIS | Genes annotated by the GO term GO:0001525. Blood vessel formation when new vessels emerge from the proliferation of pre-existing blood vessels. |
|  |  | GO: POSITIVE REGULATION OF CATALYTIC ACTIVITY | Genes annotated by the GO term GO:0043085. Any process that activates or increases the activity of an enzyme. |
|  |  | GO: ANATOMICAL STRUCTURE FORMATION | Genes annotated by the GO term GO:0048646. The process pertaining to the initial formation of an anatomical structure from unspecified parts. This process begins with the specific processes that contribute to the appearance of the discrete structure and ends when the structural rudiment is recognizable. An anatomical structure is any biological entity that occupies space and is distinguished from its surroundings. Anatomical structures can be macroscopic such as a carpel, or microscopic such as an acrosome. |
|  |  | GO: REGULATION OF MULTICELLULAR ORGANISMAL PROCESS | Genes annotated by the GO term GO:0051239. Any process that modulates the frequency, rate or extent of an organismal process, the processes pertinent to the function of an organism above the cellular level; includes the integrated processes of tissues and organs. |
|  |  | GO: REGULATION OF ANGIOGENESIS | Genes annotated by the GO term GO:0045765. Any process that modulates the frequency, rate or extent of angiogenesis. |
|  |  | GO: NEGATIVE REGULATION OF TRANSPORT | Genes annotated by the GO term GO:0051051. Any process that stops, prevents or reduces the frequency, rate or extent of the directed movement of substances (such as macromolecules, small molecules, ions) into, out of, within or between cells. |
|  |  | GO: TRANSMISSION OF NERVE IMPULSE | Genes annotated by the GO term GO:0019226. The sequential electrochemical polarization and depolarization that travels across the membrane of a nerve cell (neuron) in response to stimulation. |
|  |  | GO: ORGAN MORPHOGENESIS | Genes annotated by the GO term GO:0009887. Morphogenesis of an organ. An organ is defined as a tissue or set of tissues that work together to perform a specific function or functions. Morphogenesis is the process by which anatomical structures are generated and organized. Organs are commonly observed as visibly distinct structures, but may also exist as loosely associated clusters of cells that work together to perform a specific function or functions. |
|  |  | GO: HEART DEVELOPMENT | Genes annotated by the GO term GO:0007507. The process whose specific outcome is the progression of the heart over time, from its formation to the mature structure. The heart is a hollow, muscular organ, which, by contracting rhythmically, keeps up the circulation of the blood. |
|  |  | GO: GENERATION OF NEURONS | Genes annotated by the GO term GO:0048699. The process by which nerve cells are generated. This includes the production of neuroblasts and their differentiation into neurons. |
|  |  | GO: REGULATION OF TRANSPORT | Genes annotated by the GO term GO:0051049. Any process that modulates the frequency, rate or extent of the directed movement of substances (such as macromolecules, small molecules, ions) into, out of, within or between cells. |
|  |  | GO: NEGATIVE REGULATION OF MAP KINASE ACTIVITY | Genes annotated by the GO term GO:0043407. Any process that stops, prevents or reduces the frequency, rate or extent of MAP kinase activity. |
|  |  | GO: VASCULATURE DEVELOPMENT | Genes annotated by the GO term GO:0001944. The process whose specific outcome is the progression of the vasculature over time, from its formation to the mature structure. |
|  |  | GO: NEUROGENESIS | Genes annotated by the GO term GO:0022008. Generation of cells within the nervous system. |
|  |  | GO: NEGATIVE REGULATION OF CELL DIFFERENTIATION | Genes annotated by the GO term GO:0045596. Any process that stops, prevents or reduces the frequency, rate or extent of cell differentiation. |
|  |  | GO: REGULATION OF CELL DIFFERENTIATION | Genes annotated by the GO term GO:0045595. Any process that modulates the frequency, rate or extent of cell differentiation, the process whereby relatively unspecialized cells acquire specialized structural and functional features. |
|  |  | GO: BEHAVIOR | Genes annotated by the GO term GO:0007610. The specific actions or reactions of an organism in response to external or internal stimuli. Patterned activity of a whole organism in a manner dependent upon some combination of that organism's internal state and external conditions. |
|  |  | GO: CENTRAL NERVOUS SYSTEM DEVELOPMENT | Genes annotated by the GO term GO:0007417. The process whose specific outcome is the progression of the central nervous system over time, from its formation to the mature structure. The central nervous system is the core nervous system that serves an integrating and coordinating function. In vertebrates it consists of the brain, spinal cord and spinal nerves. In those invertebrates with a central nervous system it typically consists of a brain, cerebral ganglia and a nerve cord. |
|  |  |  |  |
| rs11564538 | PLA2G4C | GO: MEMBRANE LIPID METABOLIC PROCESS | Genes annotated by the GO term GO:0006643. The chemical reactions and pathways involving membrane lipids, any lipid found in or associated with a biological membrane. |
|  |  |  |  |
| rs11586699 | LAMC2 | KEGG: ECM RECEPTOR INTERACTION | see KEGG hsa04512 |
|  |  | KEGG: FOCAL ADHESION | see KEGG hsa04510 |
|  |  |  |  |
| rs11648430 | LAT | GO: SECOND MESSENGER MEDIATED SIGNALING | Genes annotated by the GO term GO:0019932. A series of molecular signals in which an ion or small molecule is formed or released into the cytosol, thereby helping relay the signal within the cell. |
|  |  | BioCarta: NKCELLS PATHWAY | Ras-Independent pathway in NK cell-mediated cytotoxicity |
|  |  | KEGG: NATURAL KILLER CELL MEDIATED CYTOTOXICITY | see KEGG hsa04650 |
|  |  | KEGG: FC GAMMA R MEDIATED PHAGOCYTOSIS | see KEGG hsa04666 |
|  |  | GO: REGULATION OF MULTICELLULAR ORGANISMAL PROCESS | Genes annotated by the GO term GO:0051239. Any process that modulates the frequency, rate or extent of an organismal process, the processes pertinent to the function of an organism above the cellular level; includes the integrated processes of tissues and organs. |
|  |  | KEGG: FC EPSILON RI SIGNALING PATHWAY | see KEGG hsa04664 |
|  |  |  |  |
| rs11738720 | CANX | GO: ANGIOGENESIS | Genes annotated by the GO term GO:0001525. Blood vessel formation when new vessels emerge from the proliferation of pre-existing blood vessels. |
|  |  | GO: ANATOMICAL STRUCTURE FORMATION | Genes annotated by the GO term GO:0048646. The process pertaining to the initial formation of an anatomical structure from unspecified parts. This process begins with the specific processes that contribute to the appearance of the discrete structure and ends when the structural rudiment is recognizable. An anatomical structure is any biological entity that occupies space and is distinguished from its surroundings. Anatomical structures can be macroscopic such as a carpel, or microscopic such as an acrosome. |
|  |  | GO: ORGAN MORPHOGENESIS | Genes annotated by the GO term GO:0009887. Morphogenesis of an organ. An organ is defined as a tissue or set of tissues that work together to perform a specific function or functions. Morphogenesis is the process by which anatomical structures are generated and organized. Organs are commonly observed as visibly distinct structures, but may also exist as loosely associated clusters of cells that work together to perform a specific function or functions. |
|  |  | GO: VASCULATURE DEVELOPMENT | Genes annotated by the GO term GO:0001944. The process whose specific outcome is the progression of the vasculature over time, from its formation to the mature structure. |
|  |  | GO: SECRETION | Genes annotated by the GO term GO:0046903. The regulated release of a substance by a cell, a group of cells, or a tissue. |
|  |  | GO: PROTEIN SECRETION | Genes annotated by the GO term GO:0009306. The regulated release of proteins from a cell or group of cells. |
|  |  |  |  |
| rs11999731 | PTGES | KEGG: ARACHIDONIC ACID METABOLISM | see KEGG hsa00590 |
|  |  |  |  |
| rs12202373 | ENPP1 | KEGG: NICOTINATE AND NICOTINAMIDE METABOLISM | see KEGG hsa00760 |
|  |  | KEGG: RIBOFLAVIN METABOLISM | see KEGG hsa00740 |
|  |  |  |  |
| rs12419699 | P2RX3 | GO: METAL ION TRANSMEMBRANE TRANSPORTER ACTIVITY | Genes annotated by the GO term GO:0046873. Catalysis of the transfer of metal ions from one side of a membrane to the other. |
|  |  | GO: GATED CHANNEL ACTIVITY | Genes annotated by the GO term GO:0022836. Catalysis of the transmembrane transfer of a solute by a channel that opens in response to a specific stimulus. |
|  |  | KEGG: CALCIUM SIGNALING PATHWAY | see KEGG hsa04020 |
|  |  | GO: ION CHANNEL ACTIVITY | Genes annotated by the GO term GO:0005216. Catalysis of facilitated diffusion of an ion (by an energy-independent process) by passage through a transmembrane aqueous pore or channel without evidence for a carrier-mediated mechanism. |
|  |  | GO: SUBSTRATE SPECIFIC CHANNEL ACTIVITY | Genes annotated by the GO term GO:0022838. Catalysis of energy-independent facilitated diffusion, mediated by passage of a specific solute through a transmembrane aqueous pore or channel. Stereospecificity is not exhibited but this transport may be specific for a particular molecular species or class of molecules. |
|  |  | GO: CATION CHANNEL ACTIVITY | Genes annotated by the GO term GO:0005261. Catalysis of the energy-independent passage of cations across a lipid bilayer down a concentration gradient. |
|  |  |  |  |
| rs12451873 | GOSR1 | KEGG: SNARE INTERACTIONS IN VESICULAR TRANSPORT | see KEGG hsa04130 |
|  |  | GO: ENDOSOME TRANSPORT | Genes annotated by the GO term GO:0016197. The directed movement of substances into, out of or mediated by an endosome, a membrane-bound organelle that carries materials newly ingested by endocytosis. It passes many of the materials to lysosomes for degradation. |
|  |  | GO: SECRETION | Genes annotated by the GO term GO:0046903. The regulated release of a substance by a cell, a group of cells, or a tissue. |
|  |  |  |  |
| rs12620315 | GFPT1 | GO: ALCOHOL METABOLIC PROCESS | Genes annotated by the GO term GO:0006066. The chemical reactions and pathways involving alcohols, any of a class of alkyl compounds containing a hydroxyl group. |
|  |  | GO: CELLULAR CARBOHYDRATE METABOLIC PROCESS | Genes annotated by the GO term GO:0044262. The chemical reactions and pathways involving carbohydrates, any of a group of organic compounds based of the general formula Cx(H2O)y, as carried out by individual cells. |
|  |  |  |  |
| rs12661964 | MDFI | GO: PATTERN SPECIFICATION PROCESS | Genes annotated by the GO term GO:0007389. The developmental processes that result in the creation of defined areas or spaces within an organism to which cells respond and eventually are instructed to differentiate. |
|  |  | GO: POSITIVE REGULATION OF CATALYTIC ACTIVITY | Genes annotated by the GO term GO:0043085. Any process that activates or increases the activity of an enzyme. |
|  |  | GO: NEGATIVE REGULATION OF TRANSPORT | Genes annotated by the GO term GO:0051051. Any process that stops, prevents or reduces the frequency, rate or extent of the directed movement of substances (such as macromolecules, small molecules, ions) into, out of, within or between cells. |
|  |  | GO: NEGATIVE REGULATION OF TRANSCRIPTION DNA DEPENDENT | Genes annotated by the GO term GO:0045892. Any process that stops, prevents or reduces the frequency, rate or extent of DNA-dependent transcription. |
|  |  | GO: EMBRYONIC DEVELOPMENT | Genes annotated by the GO term GO:0009790. The process whose specific outcome is the progression of an embryo from its formation until the end of its embryonic life stage. The end of the embryonic stage is organism-specific. For example, for mammals, the process would begin with zygote formation and end with birth. For insects, the process would begin at zygote formation and end with larval hatching. For plant zygotic embryos, this would be from zygote formation to the end of seed dormancy. For plant vegetative embryos, this would be from the initial determination of the cell or group of cells to form an embryo until the point when the embryo becomes independent of the parent plant. |
|  |  | GO: NEGATIVE REGULATION OF TRANSCRIPTION | Genes annotated by the GO term GO:0016481. Any process that stops, prevents or reduces the frequency, rate or extent of transcription. |
|  |  | GO: NEGATIVE REGULATION OF RNA METABOLIC PROCESS | Genes annotated by the GO term GO:0051253. Any process that stops, prevents or reduces the frequency, rate or extent of the chemical reactions and pathways involving RNA. |
|  |  | GO: REGULATION OF TRANSPORT | Genes annotated by the GO term GO:0051049. Any process that modulates the frequency, rate or extent of the directed movement of substances (such as macromolecules, small molecules, ions) into, out of, within or between cells. |
|  |  | GO: REGIONALIZATION | Genes annotated by the GO term GO:0003002. The pattern specification process by which an axis or axes is subdivided in space to define an area or volume in which specific patterns of cell differentiation will take place or in which cells interpret a specific environment. |
|  |  |  |  |
| rs12792040 | ESAM | KEGG: LEUKOCYTE TRANSENDOTHELIAL MIGRATION | see KEGG hsa04670 |
|  |  |  |  |
| rs1395468 | PLA2G2D | KEGG: FC EPSILON RI SIGNALING PATHWAY | see KEGG hsa04664 |
|  |  | GO: MEMBRANE LIPID METABOLIC PROCESS | Genes annotated by the GO term GO:0006643. The chemical reactions and pathways involving membrane lipids, any lipid found in or associated with a biological membrane. |
|  |  | KEGG: ARACHIDONIC ACID METABOLISM | see KEGG hsa00590 |
|  |  | KEGG: VASCULAR SMOOTH MUSCLE CONTRACTION | see KEGG hsa04270 |
|  |  |  |  |
| rs15251 | TCOF1 | GO: SKELETAL DEVELOPMENT | Genes annotated by the GO term GO:0001501. The process whose specific outcome is the progression of the skeleton over time, from its formation to the mature structure. The skeleton is the bony framework of the body in vertebrates (endoskeleton) or the hard outer envelope of insects (exoskeleton or dermoskeleton). |
|  |  |  |  |
| rs1528030 | NEUROD1 | GO: RNA POLYMERASE II TRANSCRIPTION FACTOR ACTIVITY | Genes annotated by the GO term GO:0003702. Functions to initiate or regulate RNA polymerase II transcription. |
|  |  |  |  |
| rs16027 | CACNA1A | GO: METAL ION TRANSMEMBRANE TRANSPORTER ACTIVITY | Genes annotated by the GO term GO:0046873. Catalysis of the transfer of metal ions from one side of a membrane to the other. |
|  |  | GO: VOLTAGE GATED CHANNEL ACTIVITY | Genes annotated by the GO term GO:0022832. Catalysis of the transmembrane transfer of a solute by a channel whose open state is dependent on the voltage across the membrane in which it is embedded. |
|  |  | GO: GATED CHANNEL ACTIVITY | Genes annotated by the GO term GO:0022836. Catalysis of the transmembrane transfer of a solute by a channel that opens in response to a specific stimulus. |
|  |  | GO: VOLTAGE GATED CATION CHANNEL ACTIVITY | Genes annotated by the GO term GO:0022843. Catalysis of the transmembrane transfer of a cation by a voltage-gated channel. A cation is a positively charged ion. |
|  |  | KEGG: CALCIUM SIGNALING PATHWAY | see KEGG hsa04020 |
|  |  | GO: ION CHANNEL ACTIVITY | Genes annotated by the GO term GO:0005216. Catalysis of facilitated diffusion of an ion (by an energy-independent process) by passage through a transmembrane aqueous pore or channel without evidence for a carrier-mediated mechanism. |
|  |  | GO: SUBSTRATE SPECIFIC CHANNEL ACTIVITY | Genes annotated by the GO term GO:0022838. Catalysis of energy-independent facilitated diffusion, mediated by passage of a specific solute through a transmembrane aqueous pore or channel. Stereospecificity is not exhibited but this transport may be specific for a particular molecular species or class of molecules. |
|  |  | KEGG: TASTE TRANSDUCTION | see KEGG hsa04742 |
|  |  | GO: CATION CHANNEL ACTIVITY | Genes annotated by the GO term GO:0005261. Catalysis of the energy-independent passage of cations across a lipid bilayer down a concentration gradient. |
|  |  | GO: VOLTAGE GATED CALCIUM CHANNEL ACTIVITY | Genes annotated by the GO term GO:0005245. Catalysis of the transmembrane transfer of a calcium ion by a voltage-gated channel. |
|  |  |  |  |
| rs17287997 | BCL2A1 | GO: NEGATIVE REGULATION OF DEVELOPMENTAL PROCESS | Genes annotated by the GO term GO:0051093. Any process that stops, prevents or reduces the rate or extent of development, the biological process whose specific outcome is the progression of an organism over time from an initial condition (e.g. a zygote, or a young adult) to a later condition (e.g. a multicellular animal or an aged adult). |
|  |  | GO: NEGATIVE REGULATION OF PROGRAMMED CELL DEATH | Genes annotated by the GO term GO:0043069. Any process that stops, prevents or reduces the frequency, rate or extent of programmed cell death, cell death resulting from activation of endogenous cellular processes. |
|  |  | GO: NEGATIVE REGULATION OF APOPTOSIS | Genes annotated by the GO term GO:0043066. Any process that stops, prevents or reduces the frequency, rate or extent of cell death by apoptosis. |
|  |  | GO: ANTI APOPTOSIS | Genes annotated by the GO term GO:0006916. A process which directly inhibits any of the steps required for cell death by apoptosis. |
|  |  |  |  |
| rs17762434 | VEZF1 | GO: CELLULAR DEFENSE RESPONSE | Genes annotated by the GO term GO:0006968. A defense response that is mediated by cells. |
|  |  | GO: RNA POLYMERASE II TRANSCRIPTION FACTOR ACTIVITY | Genes annotated by the GO term GO:0003702. Functions to initiate or regulate RNA polymerase II transcription. |
|  |  |  |  |
| rs2030922 | STX1A | GO: GENERATION OF A SIGNAL INVOLVED IN CELL CELL SIGNALING | Genes annotated by the GO term GO:0003001. The cellular process by which a physical entity or change in state, a signal, is created that originates in one cell and is used to transfer information to another cell. This process begins with the initial formation of the signal and ends with the mature form and placement of the signal. |
|  |  | KEGG: SNARE INTERACTIONS IN VESICULAR TRANSPORT | see KEGG hsa04130 |
|  |  | GO: REGULATION OF HORMONE SECRETION | Genes annotated by the GO term GO:0046883. Any process that modulates the frequency, rate or extent of the regulated release of a hormone from a cell or group of cells. |
|  |  | GO: HORMONE SECRETION | Genes annotated by the GO term GO:0046879. The regulated release of hormones, substances with a specific regulatory effect on a particular organ or group of cells. |
|  |  | GO: REGULATION OF TRANSPORT | Genes annotated by the GO term GO:0051049. Any process that modulates the frequency, rate or extent of the directed movement of substances (such as macromolecules, small molecules, ions) into, out of, within or between cells. |
|  |  | GO: SECRETION | Genes annotated by the GO term GO:0046903. The regulated release of a substance by a cell, a group of cells, or a tissue. |
|  |  |  |  |
| rs2052474 | CNN1 | GO: SMOOTH MUSCLE CONTRACTION GO 0006939 | Genes annotated by the GO term GO:0006939. A process whereby force is generated within smooth muscle tissue, resulting in a change in muscle geometry. Force generation involves a chemo-mechanical energy conversion step. The chemo-mechanical energy conversion step is carried out by the actin/myosin complex activity, which generates force through ATP hydrolysis. Smooth muscle differs from striated muscle in the much higher actin/myosin ratio, the absence of conspicuous sarcomeres and the ability to contract to a much smaller fraction of its resting length. |
|  |  | GO: REGULATION OF MUSCLE CONTRACTION | Genes annotated by the GO term GO:0006937. Any process that modulates the frequency, rate or extent of muscle contraction. |
|  |  | GO: REGULATION OF MULTICELLULAR ORGANISMAL PROCESS | Genes annotated by the GO term GO:0051239. Any process that modulates the frequency, rate or extent of an organismal process, the processes pertinent to the function of an organism above the cellular level; includes the integrated processes of tissues and organs. |
|  |  |  |  |
| rs2092109 | PIGT | GO: MEMBRANE LIPID METABOLIC PROCESS | Genes annotated by the GO term GO:0006643. The chemical reactions and pathways involving membrane lipids, any lipid found in or associated with a biological membrane. |
|  |  | GO: INTRINSIC TO ENDOPLASMIC RETICULUM MEMBRANE | Genes annotated by the GO term GO:0031227. Located in the endoplasmic reticulum membrane such that some covalently attached portion of the gene product, for example part of a peptide sequence or some other covalently attached moiety such as a GPI anchor, spans or is embedded in one or both leaflets of the membrane. |
|  |  | GO: INTEGRAL TO ENDOPLASMIC RETICULUM MEMBRANE | Genes annotated by the GO term GO:0030176. Penetrating at least one phospholipid bilayer of an endoplasmic reticulum membrane. May also refer to the state of being buried in the bilayer with no exposure outside the bilayer. |
|  |  |  |  |
| rs2146315 | HIVEP3 | GO: TRANSCRIPTION ACTIVATOR ACTIVITY | Genes annotated by the GO term GO:0016563. Any transcription regulator activity required for initiation or upregulation of transcription. |
|  |  |  |  |
| rs2166706 | MTNR1B | GO: SECOND MESSENGER MEDIATED SIGNALING | Genes annotated by the GO term GO:0019932. A series of molecular signals in which an ion or small molecule is formed or released into the cytosol, thereby helping relay the signal within the cell. |
|  |  | GO: G PROTEIN SIGNALING COUPLED TO CYCLIC NUCLEOTIDE SECOND MESSENGER | Genes annotated by the GO term GO:0007187. The series of molecular signals generated as a consequence of a G-protein coupled receptor binding to its physiological ligand, followed by modulation of a nucleotide cyclase activity and a subsequent change in the concentration of a cyclic nucleotide. |
|  |  | GO: CYCLIC NUCLEOTIDE MEDIATED SIGNALING | Genes annotated by the GO term GO:0019935. A series of molecular signals in which a cell uses a cyclic nucleotide to convert an extracellular signal into a response. |
|  |  | GO: SYNAPTIC TRANSMISSION | Genes annotated by the GO term GO:0007268. The process of communication from a neuron to a target (neuron, muscle, or secretory cell) across a synapse. |
|  |  | GO: TRANSMISSION OF NERVE IMPULSE | Genes annotated by the GO term GO:0019226. The sequential electrochemical polarization and depolarization that travels across the membrane of a nerve cell (neuron) in response to stimulation. |
|  |  | GO: RHODOPSIN LIKE RECEPTOR ACTIVITY | Genes annotated by the GO term GO:0001584. A G-protein coupled receptor that is structurally/functionally related to the rhodopsin receptor. |
|  |  |  |  |
| rs2197089 | LPL | BioCarta: PPARA PATHWAY | Mechanism of Gene Regulation by Peroxisome Proliferators via PPARa(alpha) |
|  |  | GO: HEPARIN BINDING | Genes annotated by the GO term GO:0008201. Interacting selectively with heparin, any member of a group of glycosaminoglycans found mainly as an intracellular component of mast cells and which consist predominantly of alternating alpha1-4-linked D-galactose and N-acetyl-D-glucosamine-6-sulfate residues. |
|  |  | GO: MEMBRANE LIPID METABOLIC PROCESS | Genes annotated by the GO term GO:0006643. The chemical reactions and pathways involving membrane lipids, any lipid found in or associated with a biological membrane. |
|  |  | GO: POLYSACCHARIDE BINDING | Genes annotated by the GO term GO:0030247. Interacting selectively with any polysaccharide. |
|  |  | GO: GLYCOSAMINOGLYCAN BINDING | Genes annotated by the GO term GO:0005539. Interacting selectively with any glycan (polysaccharide) containing a substantial proportion of aminomonosaccharide residues. |
|  |  |  |  |
| rs2266782 | FMO3 | GO: MICROSOME | Genes annotated by the GO term GO:0005792. Any of the small, heterogeneous, artifactual, vesicular particles, 50-150 nm in diameter, that are formed when some eukaryotic cells are homogenized and that sediment on centrifugation at 100000 g. |
|  |  |  |  |
| rs2302764 | CHRNB1 | GO: CATION TRANSPORT | Genes annotated by the GO term GO:0006812. The directed movement of cations, atoms or small molecules with a net positive charge, into, out of, within or between cells. |
|  |  | GO: SYNAPTIC TRANSMISSION | Genes annotated by the GO term GO:0007268. The process of communication from a neuron to a target (neuron, muscle, or secretory cell) across a synapse. |
|  |  | GO: ION TRANSPORT | Genes annotated by the GO term GO:0006811. The directed movement of charged atoms or small charged molecules into, out of, within or between cells. |
|  |  | GO: TRANSMISSION OF NERVE IMPULSE | Genes annotated by the GO term GO:0019226. The sequential electrochemical polarization and depolarization that travels across the membrane of a nerve cell (neuron) in response to stimulation. |
|  |  | GO: AMINE BINDING | Genes annotated by the GO term GO:0043176. Interacting selectively with any organic compound that is weakly basic in character and contains an amino or a substituted amino group. |
|  |  | GO: ACETYLCHOLINE BINDING | Genes annotated by the GO term GO:0042166. Interacting selectively with acetylcholine, an acetic acid ester of the organic base choline that functions as a neurotransmitter, released at the synapses of parasympathetic nerves and at neuromuscular junctions. |
|  |  |  |  |
| rs2303138 | LNPEP | GO: AMINOPEPTIDASE ACTIVITY | Genes annotated by the GO term GO:0004177. Catalysis of the hydrolysis of N-terminal amino acid residues from oligopeptides or polypeptides. |
|  |  |  |  |
| rs2304088 | CARM1 | BioCarta: CARM1 PATHWAY | Transcription Regulation by Methyltransferase of CARM1 |
|  |  | GO: TRANSCRIPTION ACTIVATOR ACTIVITY | Genes annotated by the GO term GO:0016563. Any transcription regulator activity required for initiation or upregulation of transcription. |
|  |  | GO: ESTABLISHMENT AND OR MAINTENANCE OF CHROMATIN ARCHITECTURE | Genes annotated by the GO term GO:0006325. The specification, formation and maintenance of the physical structure of eukaryotic chromatin. |
|  |  | GO: ONE CARBON COMPOUND METABOLIC PROCESS | Genes annotated by the GO term GO:0006730. The chemical reactions and pathways involving compounds containing a single carbon atom. |
|  |  | GO: CHROMATIN MODIFICATION | Genes annotated by the GO term GO:0016568. The alteration of DNA or protein in chromatin, which may result in changing the chromatin structure. |
|  |  | GO: METHYLTRANSFERASE ACTIVITY | Genes annotated by the GO term GO:0008168. Catalysis of the transfer of a methyl group to an acceptor molecule. |
|  |  | GO: PROTEIN METHYLTRANSFERASE ACTIVITY | Genes annotated by the GO term GO:0008276. Catalysis of the transfer of a methyl group (CH3-) to a protein. |
|  |  | GO: HISTONE MODIFICATION | Genes annotated by the GO term GO:0016570. The covalent alteration of one or more amino acid residues within a histone protein. |
|  |  | GO: TRANSCRIPTION COACTIVATOR ACTIVITY | Genes annotated by the GO term GO:0003713. The function of a transcription cofactor that activates transcription from a RNA polymerase II promoter; does not bind DNA itself. |
|  |  | GO: COVALENT CHROMATIN MODIFICATION | Genes annotated by the GO term GO:0016569. The alteration of DNA or protein in chromatin by the covalent addition or removal of chemical groups. |
|  |  | BioCarta: CARM ER PATHWAY | CARM1 and Regulation of the Estrogen Receptor |
|  |  |  |  |
| rs2364598 | SP1 | BioCarta: PPARA PATHWAY | Mechanism of Gene Regulation by Peroxisome Proliferators via PPARa(alpha) |
|  |  | GO: TRANSCRIPTION ACTIVATOR ACTIVITY | Genes annotated by the GO term GO:0016563. Any transcription regulator activity required for initiation or upregulation of transcription. |
|  |  | BioCarta: HCMV PATHWAY | Human Cytomegalovirus and Map Kinase Pathways |
|  |  |  |  |
| rs2429919 | MNT | GO: TRANSCRIPTION ACTIVATOR ACTIVITY | Genes annotated by the GO term GO:0016563. Any transcription regulator activity required for initiation or upregulation of transcription. |
|  |  | GO: TRANSCRIPTION REPRESSOR ACTIVITY | Genes annotated by the GO term GO:0016564. Any transcription regulator activity that prevents or downregulates transcription. |
|  |  | GO: TRANSCRIPTION COACTIVATOR ACTIVITY | Genes annotated by the GO term GO:0003713. The function of a transcription cofactor that activates transcription from a RNA polymerase II promoter; does not bind DNA itself. |
|  |  |  |  |
| rs2731672 | GRK6 | GO: REGULATION OF G PROTEIN COUPLED RECEPTOR PROTEIN SIGNALING PATHWAY | Genes annotated by the GO term GO:0008277. Any process that modulates the frequency, rate or extent of G-protein coupled receptor protein signaling pathway activity. |
|  |  |  |  |
| rs2791494 | CLCA1 | GO: ION CHANNEL ACTIVITY | Genes annotated by the GO term GO:0005216. Catalysis of facilitated diffusion of an ion (by an energy-independent process) by passage through a transmembrane aqueous pore or channel without evidence for a carrier-mediated mechanism. |
|  |  | GO: SUBSTRATE SPECIFIC CHANNEL ACTIVITY | Genes annotated by the GO term GO:0022838. Catalysis of energy-independent facilitated diffusion, mediated by passage of a specific solute through a transmembrane aqueous pore or channel. Stereospecificity is not exhibited but this transport may be specific for a particular molecular species or class of molecules. |
|  |  | GO: ANION CHANNEL ACTIVITY | Genes annotated by the GO term GO:0005253. Catalysis of the energy-independent passage of anions across a lipid bilayer down a concentration gradient. |
|  |  | GO: CHLORIDE CHANNEL ACTIVITY | Genes annotated by the GO term GO:0005254. Catalysis of facilitated diffusion of an chloride (by an energy-independent process) involving passage through a transmembrane aqueous pore or channel without evidence for a carrier-mediated mechanism. |
|  |  |  |  |
| rs35270239 | DMBX1 | GO: GROWTH | Genes annotated by the GO term GO:0040007. The increase in size or mass of an entire organism, a part of an organism or a cell. |
|  |  | GO: NEGATIVE REGULATION OF TRANSCRIPTION | Genes annotated by the GO term GO:0016481. Any process that stops, prevents or reduces the frequency, rate or extent of transcription. |
|  |  | GO: TRANSCRIPTION REPRESSOR ACTIVITY | Genes annotated by the GO term GO:0016564. Any transcription regulator activity that prevents or downregulates transcription. |
|  |  | GO: DEVELOPMENTAL GROWTH | Genes annotated by the GO term GO:0048589. The increase in size or mass of an entire organism, a part of an organism or a cell, where the increase in size or mass has the specific outcome of the progression of the organism over time from one condition to another. |
|  |  | GO: CENTRAL NERVOUS SYSTEM DEVELOPMENT | Genes annotated by the GO term GO:0007417. The process whose specific outcome is the progression of the central nervous system over time, from its formation to the mature structure. The central nervous system is the core nervous system that serves an integrating and coordinating function. In vertebrates it consists of the brain, spinal cord and spinal nerves. In those invertebrates with a central nervous system it typically consists of a brain, cerebral ganglia and a nerve cord. |
|  |  |  |  |
| rs3758354 | ANXA1 | GO: NEGATIVE REGULATION OF DEVELOPMENTAL PROCESS | Genes annotated by the GO term GO:0051093. Any process that stops, prevents or reduces the rate or extent of development, the biological process whose specific outcome is the progression of an organism over time from an initial condition (e.g. a zygote, or a young adult) to a later condition (e.g. a multicellular animal or an aged adult). |
|  |  | GO: NEGATIVE REGULATION OF PROGRAMMED CELL DEATH | Genes annotated by the GO term GO:0043069. Any process that stops, prevents or reduces the frequency, rate or extent of programmed cell death, cell death resulting from activation of endogenous cellular processes. |
|  |  | GO: NEGATIVE REGULATION OF APOPTOSIS | Genes annotated by the GO term GO:0043066. Any process that stops, prevents or reduces the frequency, rate or extent of cell death by apoptosis. |
|  |  | GO: ANTI APOPTOSIS | Genes annotated by the GO term GO:0006916. A process which directly inhibits any of the steps required for cell death by apoptosis. |
|  |  |  |  |
| rs3790756 | HTR6 | GO: SECOND MESSENGER MEDIATED SIGNALING | Genes annotated by the GO term GO:0019932. A series of molecular signals in which an ion or small molecule is formed or released into the cytosol, thereby helping relay the signal within the cell. |
|  |  | GO: G PROTEIN SIGNALING COUPLED TO CYCLIC NUCLEOTIDE SECOND MESSENGER | Genes annotated by the GO term GO:0007187. The series of molecular signals generated as a consequence of a G-protein coupled receptor binding to its physiological ligand, followed by modulation of a nucleotide cyclase activity and a subsequent change in the concentration of a cyclic nucleotide. |
|  |  | GO: CYCLIC NUCLEOTIDE MEDIATED SIGNALING | Genes annotated by the GO term GO:0019935. A series of molecular signals in which a cell uses a cyclic nucleotide to convert an extracellular signal into a response. |
|  |  | GO: AMINE RECEPTOR ACTIVITY | Genes annotated by the GO term GO:0008227. Combining with a biogenic amine to initiate a change in cell activity. |
|  |  | KEGG: CALCIUM SIGNALING PATHWAY | see KEGG hsa04020 |
|  |  | GO: SYNAPTIC TRANSMISSION | Genes annotated by the GO term GO:0007268. The process of communication from a neuron to a target (neuron, muscle, or secretory cell) across a synapse. |
|  |  | GO: TRANSMISSION OF NERVE IMPULSE | Genes annotated by the GO term GO:0019226. The sequential electrochemical polarization and depolarization that travels across the membrane of a nerve cell (neuron) in response to stimulation. |
|  |  | GO: RHODOPSIN LIKE RECEPTOR ACTIVITY | Genes annotated by the GO term GO:0001584. A G-protein coupled receptor that is structurally/functionally related to the rhodopsin receptor. |
|  |  |  |  |
| rs3816208 | SDC2 | KEGG: ECM RECEPTOR INTERACTION | see KEGG hsa04512 |
|  |  |  |  |
| rs4789301 | AANAT | GO: RHYTHMIC PROCESS | Genes annotated by the GO term GO:0048511. Those processes pertinent to the generation and maintenance of rhythms in the physiology of an organism. |
|  |  |  |  |
| rs4829424 | NR0B1 | GO: GLAND DEVELOPMENT | Genes annotated by the GO term GO:0048732. The process whose specific outcome is the progression of a gland over time, from its formation to the mature structure. A gland is an organ specialised for secretion. |
|  |  | GO: RIBOSOME | Genes annotated by the GO term GO:0005840. An intracellular organelle, about 200 A in diameter, consisting of RNA and protein. It is the site of protein biosynthesis resulting from translation of messenger RNA (mRNA). It consists of two subunits, one large and one small, each containing only protein and RNA. Both the ribosome and its subunits are characterized by their sedimentation coefficients, expressed in Svedberg units (symbol: S). Hence, the prokaryotic ribosome (70S) comprises a large (50S) subunit and a small (30S) subunit, while the eukaryotic ribosome (80S) comprises a large (60S) subunit and a small (40S) subunit. Two sites on the ribosomal large subunit are involved in translation, namely the aminoacyl site (A site) and peptidyl site (P site). Ribosomes from prokaryotes, eukaryotes, mitochondria, and chloroplasts have characteristically distinct ribosomal proteins. |
|  |  | GO: STEROID METABOLIC PROCESS | Genes annotated by the GO term GO:0008202. The chemical reactions and pathways involving steroids, compounds with a 1,2,cyclopentanoperhydrophenanthrene nucleus. |
|  |  | GO: NEGATIVE REGULATION OF TRANSCRIPTION DNA DEPENDENT | Genes annotated by the GO term GO:0045892. Any process that stops, prevents or reduces the frequency, rate or extent of DNA-dependent transcription. |
|  |  | GO: NEGATIVE REGULATION OF TRANSCRIPTION | Genes annotated by the GO term GO:0016481. Any process that stops, prevents or reduces the frequency, rate or extent of transcription. |
|  |  | GO: RNA POLYMERASE II TRANSCRIPTION FACTOR ACTIVITY | Genes annotated by the GO term GO:0003702. Functions to initiate or regulate RNA polymerase II transcription. |
|  |  | GO: NEGATIVE REGULATION OF RNA METABOLIC PROCESS | Genes annotated by the GO term GO:0051253. Any process that stops, prevents or reduces the frequency, rate or extent of the chemical reactions and pathways involving RNA. |
|  |  | BioCarta: CARM ER PATHWAY | CARM1 and Regulation of the Estrogen Receptor |
|  |  | GO: STRUCTURE SPECIFIC DNA BINDING | Genes annotated by the GO term GO:0043566. Interacting selectively with DNA of a specific structure or configuration e.g. triplex DNA binding or bent DNA binding. |
|  |  |  |  |
| rs4917 | AHSG | GO: SKELETAL DEVELOPMENT | Genes annotated by the GO term GO:0001501. The process whose specific outcome is the progression of the skeleton over time, from its formation to the mature structure. The skeleton is the bony framework of the body in vertebrates (endoskeleton) or the hard outer envelope of insects (exoskeleton or dermoskeleton). |
|  |  | GO: BONE REMODELING | Genes annotated by the GO term GO:0046849. The continuous turnover of bone matrix and mineral that involves first, an increase in resorption (osteoclastic activity) and later, reactive bone formation (osteoblastic activity). The process of bone remodeling takes place in the adult skeleton at discrete foci. The process ensures the mechanical integrity of the skeleton throughout life and plays an important role in calcium homeostasis. An imbalance in the regulation of bone resorption and bone formation results in many of the metabolic bone diseases, such as osteoporosis. |
|  |  | GO: TISSUE REMODELING | Genes annotated by the GO term GO:0048771. The reorganization or renovation of existing tissues. This process can either change the characteristics of a tissue such as in blood vessel remodeling, or result in the dynamic equilibrium of a tissue such as in bone remodeling. |
|  |  | GO: NEGATIVE REGULATION OF DEVELOPMENTAL PROCESS | Genes annotated by the GO term GO:0051093. Any process that stops, prevents or reduces the rate or extent of development, the biological process whose specific outcome is the progression of an organism over time from an initial condition (e.g. a zygote, or a young adult) to a later condition (e.g. a multicellular animal or an aged adult). |
|  |  | GO: REGULATION OF MULTICELLULAR ORGANISMAL PROCESS | Genes annotated by the GO term GO:0051239. Any process that modulates the frequency, rate or extent of an organismal process, the processes pertinent to the function of an organism above the cellular level; includes the integrated processes of tissues and organs. |
|  |  | GO: REGULATION OF TRANSPORT | Genes annotated by the GO term GO:0051049. Any process that modulates the frequency, rate or extent of the directed movement of substances (such as macromolecules, small molecules, ions) into, out of, within or between cells. |
|  |  | GO: MEMBRANE ORGANIZATION AND BIOGENESIS | Genes annotated by the GO term GO:0016044. A process that is carried out at the cellular level which results in the formation, arrangement of constituent parts, or disassembly of membranes inside and surrounding the cell. |
|  |  |  |  |
| rs4973003 | NMUR1 | GO: SECOND MESSENGER MEDIATED SIGNALING | Genes annotated by the GO term GO:0019932. A series of molecular signals in which an ion or small molecule is formed or released into the cytosol, thereby helping relay the signal within the cell. |
|  |  | GO: SMOOTH MUSCLE CONTRACTION GO 0006939 | Genes annotated by the GO term GO:0006939. A process whereby force is generated within smooth muscle tissue, resulting in a change in muscle geometry. Force generation involves a chemo-mechanical energy conversion step. The chemo-mechanical energy conversion step is carried out by the actin/myosin complex activity, which generates force through ATP hydrolysis. Smooth muscle differs from striated muscle in the much higher actin/myosin ratio, the absence of conspicuous sarcomeres and the ability to contract to a much smaller fraction of its resting length. |
|  |  | GO: POSITIVE REGULATION OF HYDROLASE ACTIVITY | Genes annotated by the GO term GO:0051345. Any process that activates or increases the frequency, rate or extent of hydrolase activity, the catalysis of the hydrolysis of various bonds. |
|  |  | GO: POSITIVE REGULATION OF CATALYTIC ACTIVITY | Genes annotated by the GO term GO:0043085. Any process that activates or increases the activity of an enzyme. |
|  |  | GO: CATION TRANSPORT | Genes annotated by the GO term GO:0006812. The directed movement of cations, atoms or small molecules with a net positive charge, into, out of, within or between cells. |
|  |  | GO: METAL ION TRANSPORT | Genes annotated by the GO term GO:0030001. The directed movement of metal ions, any metal ion with an electric charge, into, out of, within or between cells. |
|  |  | GO: ION TRANSPORT | Genes annotated by the GO term GO:0006811. The directed movement of charged atoms or small charged molecules into, out of, within or between cells. |
|  |  | GO: TRANSMISSION OF NERVE IMPULSE | Genes annotated by the GO term GO:0019226. The sequential electrochemical polarization and depolarization that travels across the membrane of a nerve cell (neuron) in response to stimulation. |
|  |  | GO: RHODOPSIN LIKE RECEPTOR ACTIVITY | Genes annotated by the GO term GO:0001584. A G-protein coupled receptor that is structurally/functionally related to the rhodopsin receptor. |
|  |  | GO: SECRETION | Genes annotated by the GO term GO:0046903. The regulated release of a substance by a cell, a group of cells, or a tissue. |
|  |  |  |  |
| rs569072 | RSF1 | GO: TRANSCRIPTION ACTIVATOR ACTIVITY | Genes annotated by the GO term GO:0016563. Any transcription regulator activity required for initiation or upregulation of transcription. |
|  |  | GO: ESTABLISHMENT AND OR MAINTENANCE OF CHROMATIN ARCHITECTURE | Genes annotated by the GO term GO:0006325. The specification, formation and maintenance of the physical structure of eukaryotic chromatin. |
|  |  | GO: NEGATIVE REGULATION OF TRANSCRIPTION DNA DEPENDENT | Genes annotated by the GO term GO:0045892. Any process that stops, prevents or reduces the frequency, rate or extent of DNA-dependent transcription. |
|  |  | GO: NEGATIVE REGULATION OF TRANSCRIPTION | Genes annotated by the GO term GO:0016481. Any process that stops, prevents or reduces the frequency, rate or extent of transcription. |
|  |  | GO: CHROMATIN MODIFICATION | Genes annotated by the GO term GO:0016568. The alteration of DNA or protein in chromatin, which may result in changing the chromatin structure. |
|  |  | GO: NEGATIVE REGULATION OF RNA METABOLIC PROCESS | Genes annotated by the GO term GO:0051253. Any process that stops, prevents or reduces the frequency, rate or extent of the chemical reactions and pathways involving RNA. |
|  |  | GO: TRANSCRIPTION REPRESSOR ACTIVITY | Genes annotated by the GO term GO:0016564. Any transcription regulator activity that prevents or downregulates transcription. |
|  |  |  |  |
| rs5756385 | NCF4 | KEGG: LEUKOCYTE TRANSENDOTHELIAL MIGRATION | see KEGG hsa04670 |
|  |  | GO: ELECTRON TRANSPORT GO 0006118 | Genes annotated by the GO term GO:0006118. The transport of electrons from an electron donor to an electron acceptor. |
|  |  |  |  |
| rs6089789 | OPRL1 | GO: SECOND MESSENGER MEDIATED SIGNALING | Genes annotated by the GO term GO:0019932. A series of molecular signals in which an ion or small molecule is formed or released into the cytosol, thereby helping relay the signal within the cell. |
|  |  | GO: G PROTEIN SIGNALING COUPLED TO CYCLIC NUCLEOTIDE SECOND MESSENGER | Genes annotated by the GO term GO:0007187. The series of molecular signals generated as a consequence of a G-protein coupled receptor binding to its physiological ligand, followed by modulation of a nucleotide cyclase activity and a subsequent change in the concentration of a cyclic nucleotide. |
|  |  | GO: CYCLIC NUCLEOTIDE MEDIATED SIGNALING | Genes annotated by the GO term GO:0019935. A series of molecular signals in which a cell uses a cyclic nucleotide to convert an extracellular signal into a response. |
|  |  | GO: CAMP MEDIATED SIGNALING | Genes annotated by the GO term GO:0019933. A series of molecular signals in which a cell uses cyclic AMP to convert an extracellular signal into a response. |
|  |  | GO: G PROTEIN SIGNALING COUPLED TO CAMP NUCLEOTIDE SECOND MESSENGER | Genes annotated by the GO term GO:0007188. The series of molecular signals generated as a consequence of a G-protein coupled receptor binding to its physiological ligand, followed by modulation of adenylyl cyclase activity and a subsequent change in the concentration of cyclic AMP. |
|  |  |  |  |
| rs6476878 | SLC1A1 | GO: SYNAPTIC TRANSMISSION | Genes annotated by the GO term GO:0007268. The process of communication from a neuron to a target (neuron, muscle, or secretory cell) across a synapse. |
|  |  | GO: TRANSMISSION OF NERVE IMPULSE | Genes annotated by the GO term GO:0019226. The sequential electrochemical polarization and depolarization that travels across the membrane of a nerve cell (neuron) in response to stimulation. |
|  |  | GO: AMINE TRANSPORT | Genes annotated by the GO term GO:0015837. The directed movement of amines, including polyamines, organic compounds containing one or more amino groups, into, out of, within or between cells. |
|  |  |  |  |
| rs6503534 | MED24 | GO: TRANSCRIPTION ACTIVATOR ACTIVITY | Genes annotated by the GO term GO:0016563. Any transcription regulator activity required for initiation or upregulation of transcription. |
|  |  | GO: RNA POLYMERASE II TRANSCRIPTION FACTOR ACTIVITY | Genes annotated by the GO term GO:0003702. Functions to initiate or regulate RNA polymerase II transcription. |
|  |  |  |  |
| rs6587133 | MAP2K3 | BioCarta: NTHI PATHWAY | NFkB activation by Nontypeable Hemophilus influenzae |
|  |  | GO: POSITIVE REGULATION OF CATALYTIC ACTIVITY | Genes annotated by the GO term GO:0043085. Any process that activates or increases the activity of an enzyme. |
|  |  | KEGG: FC EPSILON RI SIGNALING PATHWAY | see KEGG hsa04664 |
|  |  | BioCarta: GATA3 PATHWAY | GATA3 participate in activating the Th2 cytokine genes expression |
|  |  | BioCarta: HCMV PATHWAY | Human Cytomegalovirus and Map Kinase Pathways |
|  |  |  |  |
| rs6599088 | EIF1B | GO: REGULATION OF TRANSLATIONAL INITIATION | Genes annotated by the GO term GO:0006446. Any process that modulates the frequency, rate or extent of translational initiation. |
|  |  |  |  |
| rs6759087 | GNLY | GO: CELLULAR DEFENSE RESPONSE | Genes annotated by the GO term GO:0006968. A defense response that is mediated by cells. |
|  |  |  |  |
| rs7257503 | NUP62 | GO: NEGATIVE REGULATION OF DEVELOPMENTAL PROCESS | Genes annotated by the GO term GO:0051093. Any process that stops, prevents or reduces the rate or extent of development, the biological process whose specific outcome is the progression of an organism over time from an initial condition (e.g. a zygote, or a young adult) to a later condition (e.g. a multicellular animal or an aged adult). |
|  |  | GO: NEGATIVE REGULATION OF PROGRAMMED CELL DEATH | Genes annotated by the GO term GO:0043069. Any process that stops, prevents or reduces the frequency, rate or extent of programmed cell death, cell death resulting from activation of endogenous cellular processes. |
|  |  | GO: NEGATIVE REGULATION OF APOPTOSIS | Genes annotated by the GO term GO:0043066. Any process that stops, prevents or reduces the frequency, rate or extent of cell death by apoptosis. |
|  |  |  |  |
| rs754224 | SNAP47 | KEGG: SNARE INTERACTIONS IN VESICULAR TRANSPORT | see KEGG hsa04130 |
|  |  |  |  |
| rs754920 | FLNC | KEGG: FOCAL ADHESION | see KEGG hsa04510 |
|  |  |  |  |
| rs7736084 | HRH2 | GO: SECOND MESSENGER MEDIATED SIGNALING | Genes annotated by the GO term GO:0019932. A series of molecular signals in which an ion or small molecule is formed or released into the cytosol, thereby helping relay the signal within the cell. |
|  |  | GO: G PROTEIN SIGNALING COUPLED TO CYCLIC NUCLEOTIDE SECOND MESSENGER | Genes annotated by the GO term GO:0007187. The series of molecular signals generated as a consequence of a G-protein coupled receptor binding to its physiological ligand, followed by modulation of a nucleotide cyclase activity and a subsequent change in the concentration of a cyclic nucleotide. |
|  |  | GO: CYCLIC NUCLEOTIDE MEDIATED SIGNALING | Genes annotated by the GO term GO:0019935. A series of molecular signals in which a cell uses a cyclic nucleotide to convert an extracellular signal into a response. |
|  |  | GO: AMINE RECEPTOR ACTIVITY | Genes annotated by the GO term GO:0008227. Combining with a biogenic amine to initiate a change in cell activity. |
|  |  | KEGG: CALCIUM SIGNALING PATHWAY | see KEGG hsa04020 |
|  |  | GO: RHODOPSIN LIKE RECEPTOR ACTIVITY | Genes annotated by the GO term GO:0001584. A G-protein coupled receptor that is structurally/functionally related to the rhodopsin receptor. |
|  |  |  |  |
| rs799444 | ZMIZ2 | GO: TRANSCRIPTION ACTIVATOR ACTIVITY | Genes annotated by the GO term GO:0016563. Any transcription regulator activity required for initiation or upregulation of transcription. |
|  |  | GO: TRANSCRIPTION COACTIVATOR ACTIVITY | Genes annotated by the GO term GO:0003713. The function of a transcription cofactor that activates transcription from a RNA polymerase II promoter; does not bind DNA itself. |
|  |  |  |  |
| rs8033080 | PLA2G4F | KEGG: FC GAMMA R MEDIATED PHAGOCYTOSIS | see KEGG hsa04666 |
|  |  |  |  |
| rs8104361 | CYP4F11 | GO: ELECTRON TRANSPORT GO 0006118 | Genes annotated by the GO term GO:0006118. The transport of electrons from an electron donor to an electron acceptor. |
|  |  |  |  |
| rs854680 | CCL16 | GO: BEHAVIOR | Genes annotated by the GO term GO:0007610. The specific actions or reactions of an organism in response to external or internal stimuli. Patterned activity of a whole organism in a manner dependent upon some combination of that organism's internal state and external conditions. |
|  |  |  |  |
| rs931949 | FOSL2 | BioCarta: RANKL PATHWAY | Bone Remodelling |
|  |  |  |  |
| rs948962 | MYO7A | GO: SYNAPSE | Genes annotated by the GO term GO:0045202. The junction between a nerve fiber of one neuron and another neuron or muscle fiber or glial cell; the site of interneuronal communication. As the nerve fiber approaches the synapse it enlarges into a specialized structure, the presynaptic nerve ending, which contains mitochondria and synaptic vesicles. At the tip of the nerve ending is the presynaptic membrane; facing it, and separated from it by a minute cleft (the synaptic cleft) is a specialized area of membrane on the receiving cell, known as the postsynaptic membrane. In response to the arrival of nerve impulses, the presynaptic nerve ending secretes molecules of neurotransmitters into the synaptic cleft. These diffuse across the cleft and transmit the signal to the postsynaptic membrane. |
